# Design of Digital Mental Health Platforms for Family Member Cocompletion: Scoping Review

**DOI:** 10.2196/49431

**Published:** 2024-07-03

**Authors:** Ellen T Welsh, Jennifer E McIntosh, An Vuong, Zoe C G Cloud, Eliza Hartley, James H Boyd

**Affiliations:** 1 The Bouverie Centre School of Psychology and Public Health La Trobe University Brunswick Australia; 2 School of Psychology and Public Health La Trobe University Bundoora Australia

**Keywords:** family therapy, family, couples, eHealth, digital health, platform, platforms, e–mental health, internet interventions, psychosocial interventions, psychosocial, synthesis, review methods, review methodology, scoping, mental health, utility, design, family caregiver, caregiver, parent, child, development, cocompletion, access, accessibility, engagement, families, dyad, dyadic, user engagement, digital health, user experience, mobile phone

## Abstract

**Background:**

The COVID-19 pandemic placed an additional mental health burden on individuals and families, resulting in widespread service access problems. Digital mental health interventions suggest promise for improved accessibility. Recent reviews have shown emerging evidence for individual use and early evidence for multiusers. However, attrition rates remain high for digital mental health interventions, and additional complexities exist when engaging multiple family members together.

**Objective:**

As such, this scoping review aims to detail the reported evidence for digital mental health interventions designed for family use with a focus on the build and design characteristics that promote accessibility and engagement and enable cocompletion by families.

**Methods:**

A systematic literature search of MEDLINE, Embase, PsycINFO, Web of Science, and CINAHL databases was conducted for articles published in the English language from January 2002 to March 2024. Eligible records included empirical studies of digital platforms containing some elements designed for cocompletion by related people as well as some components intended to be completed without therapist engagement. Platforms were included in cases in which clinical evidence had been documented.

**Results:**

Of the 9527 papers reviewed, 85 (0.89%) met the eligibility criteria. A total of 24 unique platforms designed for co-use by related parties were identified. Relationships between participants included couples, parent-child dyads, family caregiver–care recipient dyads, and families. Common platform features included the delivery of content via structured interventions with no to minimal tailoring or personalization offered. Some interventions provided live contact with therapists. User engagement indicators and findings varied and included user experience, satisfaction, completion rates, and feasibility. Our findings are more remarkable for what was absent in the literature than what was present. Contrary to expectations, few studies reported any design and build characteristics that enabled coparticipation. No studies reported on platform features for enabling cocompletion or considerations for ensuring individual privacy and safety. None examined platform build or design characteristics as moderators of intervention effect, and none offered a formative evaluation of the platform itself.

**Conclusions:**

In this early era of digital mental health platform design, this novel review demonstrates a striking absence of information about design elements associated with the successful engagement of multiple related users in any aspect of a therapeutic process. There remains a large gap in the literature detailing and evaluating platform design, highlighting a significant opportunity for future cross-disciplinary research. This review details the incentive for undertaking such research; suggests design considerations when building digital mental health platforms for use by families; and offers recommendations for future development, including platform co-design and formative evaluation.

## Introduction

### Family Mental Health

Normatively, mental health disorders impacted >1 billion people worldwide in 2016 [1]. The COVID-19 pandemic brought further substantial impact on mental health, placing increased demand on mental health services [2]. Mental health is inherently relational [3,4], and family members and partners are inevitably impacted by an individual’s mental health challenges [5]. During the COVID-19 pandemic, markers of heightened family stress included rising rates of family violence [6]; increased parenting stress [7]; and observed rates of maladaptive parenting practices, including neglectful, harsh, and coercive parenting [8-10].

There is a strong evidence base for family and systemic interventions for child- and adult-focused mental health challenges. Family participation supports members of the family to safely contribute to individual recovery and improved relationships [11-13] and can be more beneficial than individual work [14-16] and family educational interventions [17]. In addition, parent involvement in interventions for childhood behavioral [18] and adolescent anxiety disorders [19] has been shown to be beneficial and contributes to positive long-term outcomes.

### Digital Mental Health

The World Health Organization has emphasized the significant potential of digital mental health interventions (DMHIs) in expanding reach and access to services [20]. Such DMHIs have shown promise in reaching underserved populations [21], leading to improved management of symptoms in individuals [22], particularly youth aged <25 years [23,24]. There is growing meta-analytic evidence for positive mental health outcomes of digitally delivered versus in-person individual treatment, for example, in the field of cognitive behavioral interventions [25]. With rapid developments in technology, research interest is expanding, with most of the literature so far focused on DMHIs for individuals. For example, a review of systematic reviews of digital interventions for mental health and well-being (with no limitations placed on population) conducted in 2021 identified 246 systematic reviews published between 2016 and 2021, all of which reviewed digitally delivered mental health interventions for individuals [26].

Beyond DMHIs designed for individuals, 2 first-generation reviews of dyadic (caregiver and care recipient) [27] and couple-targeted DMHIs [28] suggest that DMHIs can decrease barriers and improve timely access and outcomes for distressed relationships. However, research into DMHIs for *families to access together* is as yet undeveloped.

Despite growing evidence, and regardless of the population targeted, retention rates for DMHIs remain low, limiting their ultimate impact [29-32]. Among other factors, interface ease of use has been identified as a barrier to DMHI retention and engagement by individuals [25,33]. It is likely that similar (or possibly even greater) barriers for family engagement in the digital mental health space exist. Given the fundamental differences in the approach and focus for family and relational interventions when compared to interventions designed for individuals [3,34], it is likely that there are unique factors to consider when designing DMHIs for use by families. This might include considerations for individual user privacy and ways in which the platform allows multiple people to contribute to and especially cocomplete activities, such as shared goal setting. Thus, it would be ill-founded to extrapolate results from studies on DMHIs designed for use by individuals and assume similar platform interaction values for families. The need for further research specific to the design of DMHIs for family use is clear.

### Design of DMHIs for Families

Therefore, the question arises about what an effective DMHI for family use might look like. Given that computers and tablets are designed for use by individuals, DMHIs intended for cocompletion by family members may use different platform and interface features to support and sustain family engagement. No review to date has examined evidence for design and build characteristics that promote cocompletion usability, including improved engagement and accessibility.

In that light, this review aimed to synthesize the available evidence regarding the build and design characteristics that enable cocompletion and discuss reported indicators of user engagement with platforms designed for such use, namely, usability, satisfaction, acceptability, and feasibility. In the digital mental health literature, these user engagement indicators measure the ability of a platform to engage and sustain users. However, there is a notable lack of agreement on both the definition and measurement of the construct of *engagement*, which can lead to inappropriate selection, presentation, and interpretation of user engagement indicators across studies [35]. As such, a scoping review was conducted, and we adopted the definition of user engagement as outlined by Perski et al [36]: *“*Engagement with [Digital Behaviour Change Interventions] is (1) the extent (e.g. amount, frequency, duration, depth) of usage and (2) a subjective experience characterised by attention, interest and affect.”

In this scoping review, we differentiate the term “platform” from the term “intervention.” We define “platform” as the tools, infrastructure, and technical foundation behind the delivery of an intervention, including interface characteristics such as the design, layout, and delivery mode. We define “intervention” as the mental health–related content that is delivered via the platform. This review sought to understand (1) the design and functionality characteristics that enable the effective engagement with and cocompletion of a family-oriented DMHI and (2) whether these elements moderate the effect of the intervention on mental health or relational outcomes. To distinguish effective platform contributors to engagement from elements pertaining to intervention content, we selected only those platforms housing interventions of established clinical efficacy (which we defined as any intervention that had at least one study reporting a significant improvement in a mental health or relational outcome). In addition, it is expected that build characteristics may vary by population, and given that there is no uniform family composition, this review scoped platforms designed for cocompletion by any family relationship type, including couples, family subsystems, and whole families.

## Methods

### Search Strategy

To identify studies reviewing platforms delivering clinical interventions designed for cocompletion by families, a systematic search was conducted following the PRISMA (Preferred Reporting Items for Systematic Reviews and Meta-Analyses) guidelines [37]. A comprehensive electronic literature search for articles published in English was conducted in the following databases: MEDLINE, Embase, and PsycINFO via the Ovid platform; CINAHL via the EBSCOhost platform, and Web of Science. In line with developments in digital technology, studies were included if they were published in or since 2002. The search was first conducted on June 24, 2022, and additional searches were conducted on November 24, 2022; April 21, 2023; and March 15, 2024.

### Eligibility Criteria

As advised by the Joanna Briggs Institute’s guidelines for conducting scoping reviews [38], the population, concept, and context framework was used to define eligibility. Textbox 1 shows the inclusion and exclusion criteria in line with the population, concept, and context framework and contains additional study elements relevant to the eligibility criteria.

Studies were not excluded when platforms contained additional components involving practitioner (sometimes referred to in the studies as a coach, professional, therapist, or staff member) engagement. Further to the inclusion and exclusion criteria outlined in Textbox 1, platforms offering interventions that had no evidence of clinical efficacy (ie, no identified studies that reported any significant improvements in mental health or relational outcomes) were excluded. Provided that at least 1 identified study established clinical efficacy for that platform, all studies on that intervention were then included regardless of whether they reported on clinical outcomes. Platforms that met all the other inclusion criteria but without established clinical efficacy are presented in Multimedia Appendix 1.

**Inclusion and exclusion criteria detailing the population, concept, and context framework for defining eligibility criteria for scoping reviews and additional study elements.**

**Inclusion criteria**

- Population: Digital mental health interventions (DMHIs) designed for completion by at least 2 related people together
- Concept: Platform design elements of DMHIs (via a web or smartphone interface) containing some component that was intended to be completed without therapist or human intervention (ie, was self-directed by participants)
- Context: Open and included all care settings (eg, primary care and community) and all jurisdictions and geographic locations
- Study type and design: Empirical studies
- Publication date: from January 1, 2002, to March 15, 2024
- Publication language: English

**Exclusion criteria**

- Population: DMHIs designed for completion by individuals or designed for use by related people but with no activities completed together (ie, completed separately) and DMHIs where children were the focus and the parent’s role was only in assisting their child to participate
- Concept: DMHIs in which the target condition was physical illness, physical activity, and weight management and programs delivered through virtual reality devices, wearable devices, DVD, or other non–web-based approaches
- Study type and design: Nonempirical studies and gray literature (ie, non–peer-reviewed or unpublished manuscripts)

### Search and Data Extraction Methodology

A total of 3 key search constructs addressed the different elements of the research question: digital intervention, mental or relational health, and population. Results were combined using Boolean operators. The search strategies for each database can be found in Multimedia Appendix 2. The reference lists of relevant reviews were also screened for potentially relevant studies. Data extraction was completed by 2 researchers trained in systematic search methodology using a standardized template, and discrepancies were resolved through discussion between the 2 researchers. In cases in which it appeared that there could be cocompletion but it was not directly specified, the study authors were contacted, and websites were searched.

### Screening and Selection Process

Search results were downloaded into EndNote (Clarivate Analytics) [39] and imported into Covidence (Veritas Health Innovation) [40]. Duplicates were first removed in EndNote and again following import into Covidence. In total, 2 researchers screened the identified studies at the title and abstract level, with 20% being double screened. Disagreements were resolved through discussion. A total of 2 researchers screened the articles at the full-text level with 20% double screening to determine eligibility against the inclusion criteria outlined previously. Reasons for exclusion at the full-text level were recorded.

### Data Synthesis

Data were synthesized using a narrative approach. Due to high variability in the reporting of outcomes and measurements across studies, a systematic or meta-analytic approach was not possible.

The included articles were grouped by the digital platform used. Information regarding the authors, the year of publication, the country where the study took place, the population, and associated user engagement indicators was extracted. Significant differences in mental health or relational outcomes following the DMHI were indicated. Details about the platforms were extracted into a separate table. Also detailed were the intervention target; the relationship between the participants; components designed to be completed in a self-paced manner, together, individually, or with a professional; tailored components; and any additional key features. Results were categorized and synthesized based on the targeted relationship for the intervention (eg, couples or families).

## Results

### Overview

**Figure 1:**
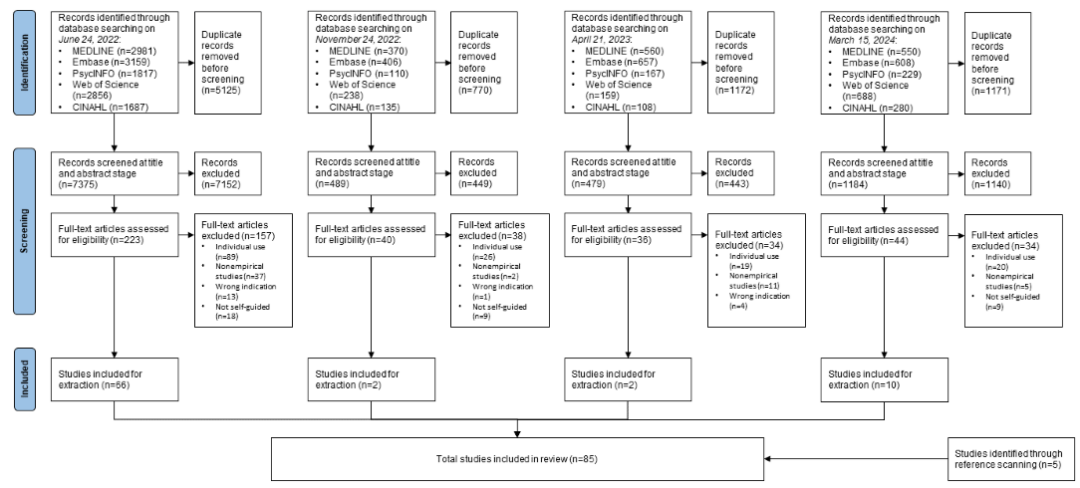
PRISMA-ScR (Preferred Reporting Items for Systematic Reviews and Meta-Analyses extension for Scoping Reviews) diagram showing the inclusion and exclusion of studies at each stage of the review process.

The combined searches yielded 17,765 results. Following removal of 46.37% (8238/17,765) of duplicates in EndNote and Covidence, 9527 papers were screened at the title and abstract level, resulting in 9184 (96.4%) exclusions. A total of 343 full-text articles were reviewed for inclusion, with 263 (76.7%) exclusions. Reasons for exclusion included the platform being designed for use by individuals (154/263, 58.6%), nonempirical studies (55/263, 20.9%), the platform not containing any self-guided components (36/263, 13.7%), or wrong indication (eg, weight loss intervention; 18/263, 6.8%). A total of 80 studies were included for data extraction. An additional 5 studies were identified through reference scanning and included in data extraction, resulting in a total of 85 studies included in this review. Figure 1 shows the PRISMA-ScR (Preferred Reporting Items for Systematic Reviews and Meta-Analyses extension for Scoping Reviews) diagram [37].

The following sections first summarize the studies identified and then report on characteristics of and findings related to the included platforms.

### Included Studies

Table 1 details the characteristics of the 85 studies, including study type, their population and sample size, usability measures and findings, and an indication of clinical efficacy based on significant improvement in mental health or relational outcomes following completion of the intervention. Among the 85 included studies, data were collected during randomized controlled trials (n=63, 74%), pilot feasibility studies (n=14, 16%), single-arm studies (n=7, 8%), and nonrandomized quasi-experimental studies (n=1, 1%).

A total of 74% (63/85) of the studies were conducted in the United States; 12% (10/85) were conducted in Canada; 5% (4/85) were conducted in Australia; 2% (2/85) were conducted in the United Kingdom; 2% (2/85) were conducted in China; and 1% (1/85) were conducted each in the Netherlands, Sweden, Japan, and Korea. In total, 52% (44/85) of the included studies were published between January 2019 and March 2024, whereas 5% (4/85) of the studies were published in the first 5 years of the search period (2002-2006 inclusive) and the remainder (37/85, 43%) were published in between these periods.

**Table 1:** Characteristics of the studies meeting the eligibility criteria, including name of the platform examined; study and country; type of study and comparator (where applicable); population, sample size, and attrition rate; relational and individual constructs or outcomes; reported user engagement indicators; and corresponding findings.

Platform, study, and country	Study characteristics		Measures or outcomes		
	Design; comparator	Population; sample size; attrition	Relational and individual	User engagement indicators	Reported findings
4Cs:CRC^a^ [41]; China	Pilot feasibility trial	Heterosexual couples where one member was experiencing colorectal cancer; 24 couples; 16.7%	Dyadic coping; cancer-related communication; self-efficacy; physical and mental health; positive and negative emotions	(1) Postintervention evaluation; (2) feasibility and acceptability	(1) Highly rated usefulness, ease of use, and satisfaction; all mean acceptability ratings >5.2/7; (2) 83.8% retention; 609 session views; mean 29 views per page; mean 3-7 page views per session per dyad
4Cs:CRC [42]; China	RCT^b^; web-based, face-to-face, blended, or control	Heterosexual couples where one member had colorectal cancer; 212 couples; 16%	Dyadic coping^c^; cancer-related communication^c^; marital satisfaction; self-efficacy^c^; physical and mental^c^ health; positive^c^ and negative^c^ emotions	Not reported	Not reported
CA-CIFFTA^d^ [43]; United Kingdom	RCT; no treatment	Hispanic (80%) and Black (20%) adolescents and their families; 80 parent-child dyads; 27% (intervention)	Family cohesion^c^; family conflict; parenting practices; adolescent behavioral problems^c^	Not reported	Not reported
C-MBI^e^ for YBCSs^f^ [44]; United States	RCT; MBI^g^ completed by YBCSs only (I-MBI^h^)	Female breast cancer survivors and their male partners; 117 couples; 26% (I-MBI) and 38% (C-MBI)	Couple functioning^c^ (I-MBI only); individual-level functioning^c^	(1) Feasibility and acceptability of YBCSs (self-report); (2) feasibility and acceptability of partners (self-report)	(1) 39% requested more contact with peers; 63% would recommend it; 77% watched all videos; 90% used the supplemental material; 91% completed some or all of the assignments; rated most useful: mindfulness sessions (80%), yoga (14%), and partner interaction (7%); time constraints were the most cited reason for not recommending the intervention; (2) 93% had no desire to interact with peers; 69% would recommend it; 69% watched all videos; 89% used the supplemental materials; 92% completed some or all of the assignments; time constraints were the most cited reason for not recommending the intervention
Cool Kids Online [45]; Australia	RCT; waitlist	Children (aged 7-12 years) with anxiety and their parents or caregivers; 95 dyads; 12% at posttreatment time point and 27% at 6-month follow-up	Anxiety diagnosis^c^; anxiety scale; life interference^c^ (parent only); mood and feelings^c^; strengths and difficulties^c^	(1) Satisfaction; (2) completion	(1) 73% of parents were satisfied or very satisfied, 92% reported it as helpful, and 97% were moderately or very confident recommending the intervention; 64% of children were “happy” with the intervention, 89% reported it as helpful or very helpful, and 70% were moderately or very confident that it would help a friend; (2) 83% accessed all lessons (mean 7.52, SD 1.23; range 3-8); received a mean of 8.8/10 (SD 1.61; range 3-10) calls
Couple HOPES^i^ [46]; Canada	Pilot feasibility trial; pretest-posttest	Couples where one member was a military member, veteran, or first responder with PTSD^j^ symptoms; 10 couples; 30%	Relationship satisfaction^c^ (partners only); conflict; PTSD symptoms^c^; partner’s accommodations to PTSD symptoms^c^; anxiety, distress, and QoL^c,k^; AOD^l^ use	Satisfaction (CSQ^m^)	PTSD: mean 3.4/4 (SD 0.7); partner: mean 3.7/4 (SD 0.4)
Couple HOPES [47]; Canada	Single arm	Couples where one member was a military member, veteran, or first responder with PTSD symptoms; 17 couples; 35%	Relationship satisfaction^c^ (ineffective arguing); PTSD self-report; partner report of PTSD symptoms; mental health; well-being^c^ (perceived health); partner accommodations	Satisfaction (CSQ)	PTSD: mean 3.5/4 (SD 0.6); partner: mean 3.7/4 (SD 0.3)
Couple HOPES [48]; Canada	Single arm	Couples where one member was a military member, veteran, or first responder with PTSD symptoms; 27 couples; 33%	Relationship functioning^c^ (ineffective arguing); mental health; well-being^c^ (perceived health and QoL)	Satisfaction (CSQ)	Partner: mean 3.7/4 (SD 0.4)
Couple HOPES [49]; United States	Pilot feasibility trial; pretest-posttest	Couples where one member was a military veteran with PTSD; 15 couples; 27%	Relationship satisfaction^c^; relationship quality^c^ (negative relationship quality); PTSD symptoms^c^; depression; QoL; significant other’s response to trauma	(1) Completion; (2) feedback	(1) Mean duration 7.20 (SD 5.56) weeks; n=11 completed; 4 noncompleters ( n=2 completed 4/7 modules, n=1 completed 2/7, and n=1 completed 1/7); n=3 “treatment responders” completed it faster; (2) coach was helpful for processing information, thoughts, and feelings; feedback videos were unrealistic or “cheesy,” others found them helpful for digesting and relating to the material
Couplelinks [50]; Canada	Pilot feasibility trial	Heterosexual couples where a member had a breast cancer diagnosis; 16 couples; 38%	Not reported	(1) Treatment satisfaction (TSQ^n^); (2) usability	(1) Mean 4/5 (SD 0.56); (2) mean 4/5 (SD 0.83)
Couplelinks [51]; Canada	Pilot feasibility trial	Heterosexual couples where a member had a breast cancer diagnosis; 6 couples; not reported	Not reported	Engagement promotion by therapist	Rational model of engagement promotion: friendly and positive yet firm approach, humanizing technology, and inclusive and empathic attitude; empirical model of engagement promotion: fostering couple-facilitator bond, fostering intervention adherence, and fostering within-couple bond
Couplelinks [52]; Canada	Pilot feasibility trial	Heterosexual couples where a member had a breast cancer diagnosis; 12 couples; not reported	Not reported	Types of engagement	Couple “types”—keen: completed with minimal engagement; compliant: met facilitator deadlines; apologetic: enjoyed it and were committed but had trouble staying on track; straggling: least engaged
Couplelinks [53]; Canada	Pilot feasibility trial	Heterosexual couples where a member had a breast cancer diagnosis; 13 couples; not reported	Not reported	Perceived benefits and limitations	58% agreed or strongly agreed that it was beneficial; 35% said that it was somewhat beneficial
Couplelinks [54]; Canada	RCT; waitlist	Heterosexual couples where a member had a breast cancer diagnosis; 67 couples; 20.5% in the intervention group and 0% in the control group	Dyadic coping^c^; dyadic consensus, cohesion, and satisfaction; marital satisfaction; collective coping^c^; anxiety^c^ and depression	Not reported	Not reported
Couplelinks [55]; Canada	RCT; waitlist	Heterosexual couples where a member had a breast cancer diagnosis; 57 participants; not reported	Not reported	Treatment satisfaction (TSQ)	Mean 4.3/5 (SD 0.54); female participants’ satisfaction ratings were significantly higher (*P*=.01); medium effect size (0.57)
eMB^o^ [56]; United States	RCT; control	Couples where one member was pregnant; 30 couples; 0%	Anxiety^c^ (pregnant person’s anxiety) and depression symptoms	(1) Satisfaction (CSQ-8^p^); (2) completion rates and adherence	(1) Excellent satisfaction: mean 3.42 (SD 0.55); pregnant: mean 3.42 (SD 0.59); and partner: mean 3.43 (SD 0.49); factors perceived to promote engagement included flexibility (independent and joint options and own pace) and focus on the self before talking to their partner; helpful elements included videos, web-based exercises, and activities; factors impacting engagement included video relatability, poor quality, outdated images, simplistic and low-technology visualizations, videos perceived as old or silly, extreme vignettes and illustrations, and videos being overly dramatized and unrelatable; (2) 50% used it alone, 9% used it together with their partner, and 27% were a combination of both; 14% did not engage; 0% completed 1 lesson per week as advised; 83 discrete log-ins; pregnant people visited more (mean 4.17 vs mean [partners] 3.44 visits to the intervention)
Embers the Dragon [57]; United Kingdom	Pilot feasibility trial; no treatment	Children aged 2-7 years and a parent; 129 families; 7.7% in the intervention group and 20.4% in the control group	Parental responses to childhood behaviors^c^	Not reported	Not reported
ePREP^q^ (studies on ePREP and OurRelationship reported separately) [58]; United States	RCT; IRC^r^	Heterosexual couples in long-term relationships; 77 couples; 0%	Commitment attitudes^c^; communication^c^; relationship satisfaction^c^; psychological aggression and assault^c^; depression, dysphoria, and well-being^c^; anxiety^c^	Level of engagement as a moderator of clinical outcomes	Higher engagement (measured via results on quizzes): greater intervention effect for alternative monitoring (β=–.33; *P*=.04), constructive communication (β=.29; *P*=.07), self-reported physical assault (β=–.58; *P*=.11), male relationship satisfaction (β=.48; *P*=.02), and female depression (β=–.37; *P*=.10). Greater time spent completing homework assignments: greater intervention effect for reported couple physical assault (β=–.69; *P*=.06), severe psychological aggression for male (β=–.90; *P*=.02) and female (β=–.09; *P*=.01) individuals, and male-perpetrated physical assault (partner report; β=–1.10; *P*=.02) but an attenuation of the positive effect of ePREP on self-reported minor psychological aggression (male individuals: β=.40 and *P*=.11; female individuals: β=.43 and *P*=.12). Male individuals with higher engagement experienced attenuation of positive impact on anxiety (β=.35; *P*=.01), and female individuals who completed more homework assignments experienced attenuation of positive impact on depression symptoms (β=.45; *P*=.03).
ePREP [59]; United States	RCT; IRC	Married couples; 52 couples; 4% after the intervention and 92% at the 1-year follow-up (8% in the intervention group and 7.6% in the control group)	Conflict resolution methods^c^; psychological aggression and assault^c^	Not reported	Not reported
FOCUS^s^ [60]; United States	Single arm; repeated measures	Patient-caregiver dyads; 38 dyads; 14%	Communication; social support; emotional distress^c^; QoL^c^; appraisal^c^; coping resources; self-efficacy	(1) Satisfaction; (2) comfort and skill using computers and the internet; (3) feasibility	(1) Ease of use: mean 6.0/7 (SD 1.1); usefulness: mean 4.4/7 (SD 1.4); general satisfaction: mean 4.8 (SD 1.7); no adverse effects of completing the intervention together; (2) moderate skill level; (3) lower enrollment rate than previous in-person RCTs (51% compared with 68%-80%); retention rate was higher than in-person RCTs (86% compared with 62%-83%)
iCBT^t^ [61]; Sweden	RCT; waitlist	Families where the child (aged 8-12 years) had a mental health diagnosis; 93 families (93 children and 182 parents); 2% in the intervention group and 4% in the control group	Anxiety^c^ (parent reported); development and well-being; child depression; primary carer mental health	(1) Satisfaction; (2) compliance	(1) Child satisfaction: mean 3.67; parent satisfaction: mean 3.78; 86% of parents agreed or very much agreed that they would recommend it; 82% of children agreed or very much agreed that the treatment was effective; (2) completed modules: mean 9.7 (SD 1.8; range 4-11); 83% completed the first 9 modules; 4 families did not complete the modules intended for both children and parents
Military Family Foundations [62]; United States	RCT; no treatment	Heterosexual couples expecting their first child where one member was in the military; 56 couples; 34.5% for mothers and 48.3% for fathers in the intervention group and 7.4% for mothers and 22.2% for fathers in the control group	Interparental relationship^c^ (mothers only); parental adjustment^c^; parent report of child outcomes^c^ (sadness)	Completion	Mean 3.93/8 completed modules
MindGuide Couple [63]; South Korea	Single arm	Korean heterosexual couples; 17 couples; 11%	Couple relationship satisfaction; family relationship^c^; mental health; positive and negative emotions; satisfaction with life^c^	(1) Satisfaction and acceptability; (2) recruitment, retention, and completion	(1) 100% reported that the content and tasks were helpful; 90% reported that the content was applicable to everyday activities; coaching was most helpful (90%), followed by video lectures (43%) and practical tasks (43%); reported benefits included flexible access (90%), being less burdensome than face-to-face interventions (86.3%), and no geographic limitations (76.7%); reported drawbacks included being too long (33.3%) and time burden (76.7%); 93.4% were satisfied; 100% were satisfied with the level of coaching; (2) 94.1% completed
MR^u^ [64]; United States	RCT; MR plus PREP^v^, PREP alone or waitlist	Veteran-partner dyads; 320 individuals (160 couples); 1.2% for MR, 2.5% for MR^c^ plus PREP, 1.2% for PREP alone, and 0% for waitlist	Perceived social support; dyadic adjustment; stress^c^; depression^c^; PTSD symptoms^c^; self-compassion^c^; response to stressful experiences^c^; sleep quality; physical pain	(1) Intervention use; (2) satisfaction	(1) Mean 2.5 hours of use per week; at 16-week follow-up: mean 90 minutes per week; (2) likely to recommend: mean (veterans) 8.7/10 and mean (partners) 9.1/10
Mother-daughter program [65]; United States	RCT; waitlist	Girls aged 10-13 years and their mothers; 202 dyads; 0% between pre- and posttest, and 2% in the intervention group and 1% in the control group lost between postintervention time point and follow-up	Mother communication^c^; conflict management^c^; daughter communication^c^; perceived rules^c^; parental monitoring; normative beliefs^c^; self-efficacy^c^; alcohol use^c^; drinking intention^c^; refusal skills; parental rules^c^; parental monitoring^c^	Anonymous program rating	Improved mother-daughter relationship: mean (girls) 4.14/5 (SD 0.35) and mean (mothers) 4.25/5 (SD 0.29); learned useful information: mean (girls) 4.16/5 (SD 0.38) and mean (mothers) 4.13/5 (SD 0.34); enjoyed the intervention: mean (girls) 4.07/5 (SD 0.39); mean (mothers) 4.20/5 (SD 0.34); found time to complete it together: mean (girls) 3.04/5 (SD 0.37); mean (mothers) 3.24/5 (SD 0.33)
Mother-daughter program [66]; United States	RCT; no treatment	Girls aged 11-13 years and their mothers; 591 dyads; 3.2% in the intervention group	Mother-daughter communication^c^; substance use^c^; family rules^c^; parental monitoring^c^; normative beliefs^c^; depression; problem-solving skills; body esteem; drug refusal self-efficacy^c^; intentions^c^	Not reported	Not reported
Mother-daughter program [67]; United States	RCT; no treatment	Girls aged 11-13 years and their mothers; 916 dyads; 5.7% from baseline to 1-year follow-up and 4.2% between 1- and 2-year follow-up	Communication^c^; mother-daughter closeness^c^; family rules^c^; parental monitoring^c^; body esteem; depression; coping ability^c^; normative beliefs^c^; refusal self-efficacy^c^; substance use^c^; intentions^c^; family rituals^c^	Not reported	Not reported
Mother-daughter program [68]; United States	RCT; no treatment	Asian American girls aged 11-14 years and their mothers; 108 dyads; 3.5% in the intervention group and 3.8% in the control group	Mother-daughter closeness^c^; mother-daughter communication^c^; substance use^c^; intentions; depression^c^; self-efficacy^c^; refusal skills^c^; parental monitoring^c^; family rules^c^	Not reported	Not reported
Mother-daughter program [69]; United States	RCT; no treatment	Asian American girls aged 11-14 years and their mothers; 108 dyads; 89.2% completed the 2-year measure	Mother-daughter closeness^c^ (girls only); mother-daughter communication^c^; parental monitoring^c^ (girls only); family rules^c^ (girls only); depressive symptoms; body esteem; self-efficacy^c^; refusal skills^c^; normative beliefs; substance use^c^; intentions^c^	Completion	96.4% completed the entire intervention; 94.6% completed the booster session; participants completed initial 9 sessions (mean 175, SD 68.9 days)
Mother-daughter program [70]; United States	RCT; no treatment	Black and Hispanic girls aged 10-13 years and their mothers; 564 dyads; 6.6% in the intervention group and 3.3% in the control group	Mother-daughter closeness; mother-daughter communication^c^; substance use^c^; normative beliefs^c^; intentions^c^; depression^c^; self-efficacy^c^; refusal skills; parental monitoring^c^; family rules^c^; body esteem	Not reported	Not reported
Mother-daughter program [71]; United States	RCT; no treatment	Mother-daughter dyads in public housing; 36 dyads; 3%	Mother-daughter closeness^c^; mother-daughter communication^c^; parental monitoring^c^; substance use; fruit and vegetable intake^c^; physical activity^c^; perceived stress^c^; drug refusal skills^c^	Fidelity	97% completed all 3 sessions
OFPS^w^ [72]; United States	Pilot feasibility trial; pretest-posttest	Children (aged 5-16 years) with moderate to severe TBI^x^ and families (all family members could participate; outcomes reported for one parent and child); 19 participants in 6 families; 0%	Child-parent relationship^c^; sibling relationship^c^; therapeutic alliance^c^	(1) Feasibility; (2) ease of use; (3) helpfulness and satisfaction (WEQ^y^)	(1) All web sessions completed without therapist assistance; families completed a mean of 10.3 web sessions; (2) ease of use: mean 3.59/5; (3) website helpfulness: mean 4.12/5; videoconferencing helpfulness: mean 4.35/5; 94.7% would recommend the intervention to others
OFPS [73]; United States	Pilot feasibility trial; pretest-posttest	Children (aged 5-16 years) with moderate to severe TBI and families (all family members could participate; outcomes reported for one parent and child); 19 participants in 6 families; 0%	Injury-related family stress and burden^c^; therapeutic alliance^c^; parental distress, depression, and anxiety^c^; child adjustment^c^	Not reported	Not reported
OFPS [74]; United States	RCT; usual care plus IRC	Children (aged 5-16 years) with moderate to severe TBI and families (all family members could participate; outcomes reported for one parent and child); 46 families; 12% in the intervention group and 0% in the IRC group	Family problem-solving, communication, and behavior management; parental problem-solving; parental distress, depression, and anxiety^c^	Website use and caregiver satisfaction (WEQ)	100% of parents indicated that they would recommend it to others; 33% indicated that they would prefer to meet in person; 94.4% reported that the website was moderately to extremely easy to use
OFPS [75]; United States	RCT; usual care plus IRC	Children (aged 5-16 years) with moderate to severe TBI and families (all family members could participate; outcomes reported for one parent and child); 46 families; 12% in the intervention group and 0% in the IRC group	Child adjustment^c^ (self-control and compliance only)	(1) Child’s self-reported website use; (2) satisfaction (WEQ)	(1) Strong negative correlations between number of sessions completed and child behavioral problems (–0.59) and parental distress (–0.60) at baseline, suggesting families with more problems at baseline completed fewer sessions; (2) 88% rated the website as at least moderately easy to use; 26% rated it as hardly or not easy to use relative to other sites; all children rated the website content as at least moderately helpful; 94% reported feeling at least moderate support and understanding when using the website; 31% reported feeling angry when using the website; 25% reported feeling moderately to extremely worried when using the website
OFPS [76]; United States	RCT; usual care plus IRC	Children (aged 5-16 years) with moderate to severe TBI and families (all family members could participate; outcomes reported for one parent and child); 46 families; 12% in the intervention group and 0% in the IRC group	Therapeutic alliance (no moderation by previous technology use); parental depression (moderated by previous technology use) and anxiety	(1) Parents’ self-reported website use; (2) satisfaction (WEQ); (3) previous computer use; (4) computer equipment comfort rating	(1) Both groups reported spending equivalent amounts of time on the website; (2) satisfaction did not differ by previous technology use; (3) significant effect of technology at home for improvements in depression (t_22_=2.24; *P*=.04); trend in the same direction for anxiety; non–technology users more likely to miss sessions (mean 16.33 missed sessions, SD 11.29; t_18_=2.43; *P*=.03); (4) technology users became more comfortable with the technology over time
OFPS [77]; United States	Pilot feasibility study; pretest-posttest	Teenagers (aged 11-18 years) with moderate to severe TBI and families (all family members could participate; outcomes reported for one parent and child only); 9 families; 0%	Family functioning^c^; adolescent adjustment; parental distress and depression^c^	Feasibility	All families completed the 10 core sessions; 6 families completed one or more supplemental sessions
OFPS [78]; United States	Pilot feasibility study; pretest-posttest	Teenagers (aged 11-18 years) with moderate to severe TBI and families (all family members could participate; outcomes reported for one parent and child only); 9 families; 0%	Not reported	(1) Self-reported website use; (2) satisfaction (WEQ and OSS^z^)	(1) In addition to parents and teenagers, 9 siblings participated in at least some of the sessions; (2) father satisfaction was generally high; 4/9 teenagers and 2/7 mothers reported a preference for face-to-face meetings; feedback provided support for acceptability and helpfulness of the intervention
OFPS [79]; United States	RCT; usual care plus IRC	Teenagers (aged 11-18 years) with moderate to severe TBI and families (all family members could participate; outcomes reported for one parent and child only); 41 families; 20% in the intervention group and 5% in the IRC group	Executive functioning^c^ (teenagers with severe TBI)	Not reported	Not reported
OFPS [80]; United States	RCT; usual care plus IRC	Teenagers (aged 11-18 years) with moderate to severe TBI and families (all family members could participate; outcomes reported for one parent and child only); 41 families; 20% in the intervention group and 5% in the IRC group	Family conflict^c^; adolescent adjustment	Self-reported website use and satisfaction	Families completed an average of 10 sessions; 95% completed all 10 sessions; 87% of parents reported meeting their goals, learning ways to improve their child’s behavior, and understanding their child better (*P*<.05 relative to IRC)
OFPS [81]; United States	RCT; usual care plus IRC	Teenagers (aged 11-18 years) with moderate to severe TBI and families (all family members could participate; outcomes reported for one parent and child only); 41 families; 20% in the intervention group and 5% in the IRC group	Parental distress and depression^c^ (lower SES^aa^ only); social problem-solving^c^ (lower SES only)	Website use, ease of use, and satisfaction (WEQ and OSS)	93% rated it as moderately or extremely helpful compared to other sites; parents’ suggestions for change included fewer questionnaires; 20% of parents agreed that the intervention was too short
OFPS [82]; United States	RCT; usual care plus IRC	Children (aged 12-17 years) with moderate to severe TBI and families (all family members could participate; outcomes reported for one parent and child only); 132 children and their families; 12.3% in the intervention group and 5.9% in the control group	Teenager executive function^c^ (older adolescents)	Not reported	Not reported
OFPS [83]; United States	RCT; usual care plus IRC	Children (aged 12-17 years) with moderate to severe TBI and families (all family members could participate; outcomes reported for one parent and child only); 132 children and their families; 12.3% in the intervention group and 5.9% in the control group	Caregiver depression and distress^c^ (intention-to-treat analysis); caregiver efficacy	(1) Previous technology use; (2) completion	(1) Previous computer use did not moderate reductions in depression and distress; nonfrequent computer users in the intervention group reported significantly higher levels of caregiver efficacy (*F*_41_=7.15; *P*=.01); (2) 43% of parents reported spending <30 minutes per week on CAPS^a^^b^; 50% reported spending 30 minutes-2 hours per week; 88% completed ≥4 sessions
OFPS [84]; United States	RCT; usual care plus IRC	Children (aged 12-17 years) with moderate to severe TBI and families (all family members could participate; outcomes reported for one parent and child only); 132 children and their families; 12.3% in the intervention group and 5.9% in the control group	Child behavioral outcomes^c^ (older adolescents)	Completion	43% of parents reported spending <30 minutes per week on CAPS; 50% reported spending 30 minutes-2 hours per week; 88% completed ≥4 sessions; 93% rated the website as moderately to extremely helpful
OFPS [85]; United States	RCT; usual care plus IRC	Children (aged 12-17 years) with moderate to severe TBI and families (all family members could participate; outcomes reported for one parent and child only); 132 children and their families; 12.3% in the intervention group and 5.9% in the control group	Parent-teenager conflict; parent-teenager interactions; structural, organizational, and transactional characteristics of families	Completion	43% of parents reported spending <30 minutes per week on CAPS; 50% reported spending 30 minutes-2 hours per week; 88% completed ≥4 sessions
OFPS [86]; United States	RCT; usual care plus IRC	Children (aged 12-17 years) with moderate to severe TBI and families (all family members could participate; outcomes reported for one parent and child only); 132 children and their families; 12.3% in the intervention group and 5.9% in the control group (final assessment: 13.4% in the intervention group and 11.4% in the control group)	Long-term caregiver depression and distress^c^ (distress only); long-term perceived parenting efficacy	Not reported	Not reported
OFPS [87]; United States	RCT; usual care plus IRC	Children (aged 12-17 years) with moderate to severe TBI and families (all family members could participate; outcomes reported for one parent and child only); 132 children and their families; 12.3% in the intervention group and 5.9% in the control group (final assessment: 30.8% in the intervention group and 19.4% in the control group)	Long-term child behavioral outcomes^c^ (internalizing behaviors of older adolescents)	Completion	Number of sessions completed unrelated to improvements in internalizing symptoms over time; those who completed more sessions reported less improvement in externalizing symptoms over time (*P*=.007)
OFPS [88]; United States	RCT; usual care plus IRC	Children (aged 12-17 years) with moderate to severe TBI and families (all family members could participate; outcomes reported for one parent and child only); 132 children and their families; 25% in the intervention group and 21% in the control group	Adolescent emotional and behavioral functioning; adolescent mood and behavior (as a function of parent marital status)	Not reported	Not reported
OFPS [89]; United States	RCT; face-to-face F-PST^a^^c^, therapist-guided F-PST, or self-guided web-based F-PST	Adolescents (aged 14-18 years) with moderate to severe TBI and families (all family members could participate; outcomes reported for one parent and child only); 149 parents and caregivers; 18%	Parent depression^c^ (therapist-guided group only); parent psychological distress^c^ (therapist-guided group only)	Computer use before and during	Parents with less comfort with technology improved more with therapist-guided treatment when compared to self-guided treatment (*F*_1,107_=3.80; *P*=.05)
OFPS [90]; United States	RCT; face-to-face F-PST, therapist-guided F-PST, or self-guided web-based F-PST	Adolescents (aged 14-18 years) with moderate to severe TBI and families (all family members could participate; outcomes reported for one parent and child only); 149 parents and caregivers; at the 9-month assessment: 35.3% in the face-to-face group, 21.5% in the therapist-guided group, and 20% in the self-guided group	Behavioral outcomes	(1) Patient-perceived preference for treatment (before the intervention); (2) adherence; (3) satisfaction; (4) computer use	(1) 71% of parents agreed or strongly agreed that self-guided F-PST was most convenient; 54% of parents agreed or strongly agreed that self-guided and therapist-guided web-based F-PST would be most beneficial; 55% of teenagers agreed or strongly agreed that self-guided F-PST was most convenient; (2) median 5 hours per week; parents assigned to their preferred group completed a mean of 5.29 sessions, and those assigned to their nonpreferred group completed a mean of 6.37 sessions; adolescents in their preferred group completed a mean of 6.12 sessions, and those in their nonpreferred group completed a mean of 5.17 sessions; adolescent treatment preference was significantly related to attrition (χ^2^=4.2, 95% CI 1.03–5.44; *P*=.04); (3) parents in the face-to-face group rated the intervention more favorably than those in the therapist-guided (Cohen *d*=0.67, 95% CI 0.10-1.15; *t*=–2.49; *P*<.04) or self-guided (Cohen *d*=1.18, 95% CI 0.56-1.62; *t*=–4.36; *P*<.001) group; parents in the face-to-face group reported higher satisfaction than parents in the self-guided group (Cohen *d*=0.63, 95% CI 0.09-1.11; *t*=–2.51; *P*=.04); (4) no significant association with treatment preference
OFPS [91]; United States	RCT; face-to-face F-PST, therapist-guided F-PST, or self-guided web-based F-PST	Adolescents (aged 14-18 years) with moderate to severe TBI and families (all family members could participate; outcomes reported for one parent and child only); 149 parents and caregivers; at the 9-month assessment: 35.3% in the face-to-face group, 21.5% in the therapist-guided group, and 20% in the self-guided group	Adolescent QoL^c^; brain injury symptoms	Not reported	Not reported
OFPS [92]; United States	RCT; TOPS^a^^d^ with family, TOPS with teenagers only, or IRC	Teenagers (aged 11-18 years) with moderate to severe TBI and families (all family members could participate; outcomes reported for one parent and child only); 152 teenagers and their families; 31% in the TOPS with family group, 24% in the TOPS with teenagers only group, and 23% in the IRC group	Child behavioral outcomes^c^ (TOPS with family)	Completion	Completion: mean sessions completed (TOPS with family) 8.00 (SD 2.90) and mean sessions completed (TOPS with teenagers only) 8.40 (SD 2.80); completed supplemental sessions: 14.29% for TOPS with family and 13.46% for TOPS with teenagers only
OFPS [93]; United States	RCT; TOPS with family, TOPS with teenagers only, or IRC	Teenagers (aged 11-18 years) with moderate to severe TBI and families (all family members could participate; outcomes reported for one parent and child only); 152 teenagers and their families; 31% in the TOPS with family group, 24% in the TOPS with teenagers only group, and 23% in the IRC group	Family functioning; family cohesion^c^ (TOPS with family and 2-parent households); parent-adolescent conflict; parental psychological distress and depression^c^ (TOPS with family and 2-parent households)	Not reported	Not reported
OurRelationship [94]; United States	RCT; waitlist	Heterosexual couples; 300 couples; 8%	Relationship satisfaction^c^; positive and negative relationship quality^c^ (reducing negative relationship quality); relationship confidence^c^; depression^c^; anxiety^c^; perceived health^c^; work functioning^c^; QoL^c^	(1) Evaluation (Client Evaluation of Services Questionnaire); (2) completion rates; (3) coach engagement	(1) Mean 26.81 (SD 4.44), nearly equivalent to in-person individual therapy (Cohen *d*=–0.07) and high-quality couple therapy (Cohen *d*=–0.18); 94% were mostly or very satisfied with the services received; 97% would recommend it to a friend; (2) 86% completed the entire intervention; an additional 5% completed up to the “Understand” phase; (3) coaches spent a mean of 51.32 (SD 17.11) minutes with the couples; individuals received a mean of 5.11 (SD 1.7) scripted chat reminders and no tailored chat messages
OurRelationship [95]; United States	RCT; waitlist	Heterosexual couples; 300 couples; 8%	Relationship satisfaction^c^ (no moderation by LI-IPV^a^^e^)	Not reported	Not reported
OurRelationship [96]; United States	RCT; waitlist	Heterosexual couples; 300 couples; 8%	Relationship satisfaction; relationship confidence; positive and negative relationship quality^c^ (moderated by rurality); depression; anxiety; perceived health^c^ (moderated by race); work functioning; QoL	(1) Evaluation (Client Evaluation of Services Questionnaire); (2) participant predictors of completion	(1) Couples were generally satisfied with the intervention (mean 26.81, SD 4.44); service evaluation was not moderated by race, ethnicity, income, educational level, or rural status; (2) Hispanic couples (OR^af^ 0.24; *P*=.009; Cohen *d*=0.79) and low-income couples (OR 0.21; *P*=.002; Cohen *d*=0.85) were more likely to drop out
OurRelationship [97]; United States	RCT; waitlist	Heterosexual couples; 300 couples; 8%	Long term: relationship satisfaction; positive^c^ and negative relationship quality; relationship confidence^c^ (Hispanic couples); depression^c^; anxiety^c^; perceived health^c^; work functioning^c^; QoL^c^	Not reported	Not reported
OurRelationship [98]; United States	RCT; waitlist	Heterosexual couples; 300 couples; 8%	Relationship satisfaction; coparenting conflict^c^ (not maintained at follow-up); child functioning^c^	Not reported	Not reported
OurRelationship [99]; United States	RCT; waitlist	Heterosexual couples; 300 couples; 8%	Relationship satisfaction^c^; communication^c^; emotional intimacy^c^; relationship problem confidence^c^; relationship problem acceptance^c^; self-protective orientation^c^	Not reported	Not reported
OurRelationship [100]; United States	RCT; waitlist	Heterosexual couples; 300 couples; 8%	Relationship satisfaction^c^ (moderated by neuroticism); relationship confidence^c^; depression (moderated by neuroticism and conscientiousness); personality	Not reported	Not reported
OurRelationship [101]; United States	RCT; low coach support or high coach support	Heterosexual couples; 356 couples; 34% in the group with high coach support and 64% in the group with low coach support	Relationship satisfaction^c^ (both groups); depression^c^ (both groups); anxiety^c^ (both groups; significantly greater in the high-support group)	Platform predictors of completion	Participants in the high-support group were significantly more likely to complete the entire intervention (66% vs 36%; χ^2^_1_=32.8, *P*<.001); participants in the high-support group were more likely to complete two-thirds of the intervention (69% vs 45%; *χ*^2^_1_=20.4, *P*<.001); no significant differences in first phase completion; completion did not differ by race, ethnicity, or household income
OurRelationship [102]; United States	RCT; low coach support, high coach support, or no coach support	Heterosexual couples; 529 couples; 93.9% in the group with no coach support, 34% in the group with high support, and 64% in the group with low support	Relationship satisfaction; relationship confidence; depression; anxiety	Platform predictors of completion	6.1% of participants in the group with no coach, 66.1% of participants in the high-support group, and 36% of participants in the low-support group completed the intervention; substantial and immediate dropout when compared with the high-support (*b*=–2.68; SE 0.35; *t*=–7.65; OR 0.07, 95% CI 0.04-0.14; *P*<.001) and low-support (*b*=–1.98; SE 0.34; *t*=–5.76; OR 0.14, 95% CI 0.07-0.27; *P*<.001; neither was significant) groups; Hispanic individuals were less likely to complete the intervention without a coach than non-Hispanic individuals (*b*=–3.99; *P*<.001); higher levels of depressive symptoms predicted less drop-off with no coach (*b*=0.08; *P*=.04)
OurRelationship [103]; United States	RCT; brief OurRelationship with coach, brief OurRelationship without coach, or waitlist	Heterosexual couples; 104 couples; 40.4% at midintervention, 25% at end of intervention, and 17.4% at follow-up in the arm with a coach and 56% at midintervention, 26% at end of intervention, and 26% at follow-up in the arm without a coach	Relationship satisfaction; positive and negative relationship quality^c^ (positives); relationship confidence; communication; anxiety; depression; perceived health and QoL; work functioning	Platform predictors of completion	Dropout rate was 9.3% for the full OurRelationship and 28.8% for the brief OurRelationship with a coach (*χ*^2^=12.1; *P*<.001); 71.2% completion in the coach condition and 42.3% completion in the no-coach condition (*χ*^*2*^*=8.8*; *P*=.003)
OurRelationship [104]; United States	Pilot; pretest-posttest	Veterans and their partners; 13 couples; 15%	Relationship satisfaction and distress; relationship conflict; depression symptoms; probable PTSD; QoL	(1) Intervention satisfaction (CSQ-8); (2) completion	(1) Mean (veterans) 3.4/4 (SD 0.4) and mean (partners) 3.2/4 (SD 0.6); 91% were mostly or very satisfied; 96% would recommend it; positive qualitative feedback included structure, videos of similar couples, and reminder calls; negative qualitative feedback included repetition, length of some content, and technical and logistic frustrations; couples preferred the coach calls; (2) completion rate was 85%; median completion time was 52 (range 29-73) days; couples received clinical contact ranging from 52 to 95 minutes in total
OurRelationship [105]; United States	Single arm; pretest-posttest	Coparenting couples; 136 couples; 20%	Relationship satisfaction and distress^c^; coparenting satisfaction^c^; gatekeeping and gate closing behaviors^c^; perception of partner’s gatekeeping and gate closing behaviors^c^	Not reported	Not reported
OurRelationship [106]; United States	RCT; OurRelationship or OurRelationship+ with greater therapist engagement	Couples; 314 couples; 64.3%	Individual use, joint use and perception of partner’s ^c^ pornography use; arguments surrounding self-, joint, and partner’s pornography consumption; individual pornography use^c^; problematic pornography use; lifestyle changes due to the COVID-19 pandemic	Not reported	Not reported
OurRelationship [107]; United States	RCT; full coach, automated coach, contingent coach, or waitlist	Couples; 740 couples; 30%	Relationship satisfaction^c^ (comparable across all types of coach support)	Completion	Completion comparable across all conditions; posterior distributions indicated that the probability of full-coach couples having higher odds of completing phases 1, 2, and 3 relative to automated-coach couples was 28.4%, 43.9%, and 77.4%, respectively; probability of full-coach couples having higher odds of completing phases 1, 2, and 3 relative to contingent-coach couples was 65%, 70%, and 92.7%, respectively; probability of contingent-coach couples having higher odds of completing phases 1, 2, and 3 relative to automated-coach couples was 15.6%, 22.6%, and 21.7%, respectively
OurRelationship and ePREP [108]; United States	RCT; OurRelationship, ePREP, or waitlist	Romantic couples; 742 couples; 10.3% at posttreatment time point, 12.5% at 2-month follow-up, and 13% at 4-month follow-up	Relationship satisfaction^c^; communication conflict^c^; emotional support^c^; intimate partner violence^c^; breakup potential^c^	(1) Evaluation (Client Evaluation of Services Questionnaire); (2) completion	(1) Participants rated the intervention positively (mean 9.9/11); 96% would recommend it to a friend; 93% were satisfied; no significant difference between OurRelationship and ePREP in satisfaction (*b*=–0.058; SE 0.148; *P*=.70); (2) 69% in both ePREP and OurRelationship completed all content
OurRelationship and ePREP [109]; United States	RCT; OurRelationship, ePREP or waitlist	Romantic couples; 742 couples; 10.3% at posttreatment time point, 12.5% at 2-month follow-up, and 13% at 4-month follow-up	Relationship satisfaction^c^; breakup potential^c^; negative communication^c^; positive communication^c^; relationship problem intensity^c^; relationship problem confidence^c^; emotional support^c^	Not reported	Not reported
OurRelationship and ePREP [110]; United States	RCT; OurRelationship, ePREP, or waitlist	Romantic couples; 742 couples; 10.3% at posttreatment time point, 12.5% at 2-month follow-up, and 13% at 4-month follow-up	Psychological distress^c^; perceived stress^c^; anger^c^; problematic alcohol use^c^; perceived health^c^; insomnia^c^; exercise^c^	Not reported	Not reported
OurRelationship and ePREP [111]; United States	RCT; OurRelationship, ePREP, or waitlist	Romantic couples; 742 couples; 10.3% at posttreatment time point, 12.5% at 2-month follow-up, and 13% at 4-month follow-up	Cooperative parenting; parenting stress; parenting nurturance^c^ (OurRelationship); physical and harsh verbal discipline^c^ (OurRelationship)	Not reported	Not reported
OurRelationship and ePREP [112]; United States	RCT; OurRelationship, ePREP, or waitlist	Romantic couples; 742 couples; 10.3% at posttreatment time point, 12.5% at 2-month follow-up, 13% at 4-month follow-up, and 18.6% at 12-month follow-up	Long term: relationship satisfaction^c^; breakup potential^c^; positive communication^c^; communication conflict^c^; emotional support^c^; intimate partner violence; psychological distress^c^; perceived stress^c^; anger^c^; alcohol use^c^; perceived health^c^; insomnia^c^	Not reported	Not reported
OurRelationship and ePREP [113]; United States	RCT; OurRelationship, ePREP, or waitlist	Military and nonmilitary couples; 90 military couples; 43% for military couples	Relationship satisfaction^c^; communication conflict^c^; emotional support^c^; breakup potential^c^; intimate partner violence; psychological distress; perceived stress; anger; substance use; perceived health	(1) Evaluation (Client Evaluation of Services Questionnaire); (2) completion	(1) Evaluation ratings were similarly positive (*b*=0.470; *P*=.07); (2) 57% of military couples completed the entire intervention (compared with 71% of civilian couples), 8% completed two-thirds, 18% completed one-third, and 18% completed none
OurRelationship and ePREP [114]; United States	RCT; OurRelationship, ePREP, or waitlist	Low-income couples; 671 couples; 36% for OurRelationship and 31% for ePREP	Relationship satisfaction^c^; communication conflict^c^; emotional support^c^; intimate partner violence; breakup potential^c^ (not maintained long term for ePREP)	(1) Evaluation (Client Evaluation of Services Questionnaire); (2) completion	(1) Participants’ satisfaction: mean (OurRelationship) 9.51/11; mean (ePREP) 9.6/11; >95% of participants indicated that the intervention helped them; 97% indicated that they would recommend the intervention; 90% were satisfied with the intervention; no reliable differences in satisfaction between the 2 interventions (*B*=0.07, 95% CI –0.07 to 0.21); (2) 64% completed OurRelationship, and 69% completed ePREP
OurRelationship and ePREP [115]; United States	RCT, OurRelationship, ePREP, or waitlist	Low-income perinatal couples; 180 couples; 32.8% for OurRelationship and 36.1% for ePREP	Relationship satisfaction^c^; perceived likelihood of breakup^c^; communication conflict^c^; sexual intimacy^c^; emotional support^c^; experience of intimate partner violence; psychological distress^c^; perceived stress^c^ (OurRelationship only)	Not reported	Not reported
OurRelationship and ePREP [116]; United States	RCT; OurRelationship, ePREP, or waitlist	Low-income couples; 659 couples; 16.8%	Relationship satisfaction^c^	Not reported	Not reported
OurRelationship and ePREP [117]; United States	RCT; OurRelationship, ePREP, or waitlist	Low-income couples; 615 couples; not reported	Perceived gratitude from partner^c^; relationship satisfaction^c^; relationship instability^c^; communication skills^c^; destructive communication^c^; partner emotional support^c^	Not reported	Not reported
ParentSTRONG [118]; United States	RCT; waitlist	Early adolescent male individuals and a parent or guardian; 119 dyads; 8.5%	Dating violence behaviors^c^; parent-child communication^c^; attitudes supporting dating violence; aggression; emotional regulation^c^	Acceptability and fidelity	90% of families completed all 6 modules; 87% of parents rated helpfulness as >4/5, and 99% of parents rated helpfulness as >3/5; 65% of teenagers rated helpfulness as >4/5, and 96% of teenagers rated helpfulness as >3/5; intervention did not allow participants to progress without completing all activities
PACT^a^^g^ [119] Australia	RCT; waitlist	Parent-child dyads in which the child (aged 2-10 years) had cerebral palsy; 67 dyads; 24.4%	Emotional availability^c^; child involvement^c^; QoL^c^; parental mindfulness^c^; parental acceptance^c^; adjustment	Not reported	Not reported
ParentWorks [120]; Australia	Single arm; pretest-posttest measures	Parent or caregiver of a child aged 2-16 years; 388 families; 92.7% (nonstarters included)	Dysfunctional parenting^c^; interparental conflict^c^; child behavioral difficulties^c^; parental mental health^c^	Satisfaction (CSQ)	Mean 5.49 (SD 0.95); no significant sex differences (t_452_=0.41; *P*>.05), indicating that mothers and fathers were equally satisfied
ParentWorks [121]; Australia	Single arm; pretest-posttest measures	Parent or caregiver of a child aged 2-16 years; 388 families; 92.7% (nonstarters included)	Parent and family functioning; parenting conflict; child behavioral difficulties; parental mental health	(1) Completion; (2) dropout characteristics; (3) participant predictors of completion	(1) For partial completers, mean 2.4/5 (SD 1.2) modules completed; for full completers, mean 5.58/6 (SD 0.76) modules completed (including 1 optional module); (2) mothers in the full completer and partial completer groups reported higher levels of conduct problems than nonstarters *F*_2,1749_=3.99; *P*<.05); (3) relative to full completers, nonstarters were more likely to have older children, be married or in a de facto relationship, have higher levels of psychological difficulties, and have lower levels of child conduct problems; relative to full completers, partial completers were more likely to be married or in a de facto relationship and have higher levels of dysfunctional parenting
PERC^a^^h^ [122]; United States	Single arm; pretest-posttest	Couples where one member had a prostate cancer diagnosis; 26 couples; 15%	Dyadic communication; relationship satisfaction; QoL^c^; symptom distress^c^; general symptoms^c^	(1) Feasibility and acceptability; (2) web activity; (3) ease of use	(1) 96% completed the intervention; (2) 37% of couples always logged in together, and 23% always logged in individually; mean 3.64 (SD 1.68) log-ins per couple; mean time spent on the platform per couple: 56.96 (SD 39.74) minutes; 83% used audio-enhanced slides; 94% visited the assignment and exercise section; (3) participants rated PERC as easy to use, engaging, and of high quality
Resilient Living [123]; the Netherlands	Pilot feasibility trial	Patients with stroke or brain tumor and their caregivers; 16 participants; 68.75%	Dyadic coping; resilience; stress; caregiver role overload; QoL; fatigue^c^; physical function^c^; anxiety^c^; sleep	(1) Intervention evaluation; (2) WiWi^a^^i^	(1) Mean 2.6/5 for “Do you think the skills you learned enhanced your resilience?” and mean 4.4/5 for “did you find the online intervention easy to use?”; remaining mean scores ranged between 3.3 and 4.2/5; length of modules and ability to complete them in their own time were identified as facilitators to use; finding time to complete them as a dyad was challenging; (2) 4/5 indicated that it was worthwhile participating in the study, 4/5 indicated that it was as expected, and 1 indicated it was better than expected
Web-based partnership support program [124]; Japan	Quasi-experimental design (nonrandomized); control	Infertile couples; 151 couples; 20.4%	QoL^c^; distress	Not reported	Not reported
Web-based PREP program [125]; United States	RCT; IRC	Heterosexual foster or adoptive couples; 32 couples; 35%	Negative communication; knowledge acquisition^c^; use of PREP skills^c^	Intervention feedback	Participants responded favorably to the intervention

^a^4Cs:CRC: Caring for Couples Coping With Colorectal Cancer.

^b^RCT: randomized controlled trial.

^c^Indicates significance, or that the intervention was superior to the comparator, at the postintervention time point for the outcome measure.

^d^CA-CIFFTA: Computer-Assisted, Culturally Informed, and Flexible Family-Based Treatment for Adolescents.

^e^C-MBI: couple mindfulness-based intervention.

^f^YBCS: young breast cancer survivor.

^g^MBI: mindfulness-based intervention.

^h^I-MBI: mindfulness-based intervention for individuals.

^i^HOPES: Helping Overcome Posttraumatic Stress Disorder and Enhance Satisfaction.

^j^PTSD: posttraumatic stress disorder.

^k^QoL: quality of life.

^l^AOD: alcohol and other drug.

^m^CSQ: Client Satisfaction Questionnaire.

^n^TSQ: Treatment Satisfaction Questionnaire.

^o^eMB: mothers and babies online course.

^p^CSQ-8: 8-item Client Satisfaction Questionnaire.

^q^ePREP: computer-based Prevention and Relationship Enhancement Program.

^r^IRC: internet resource comparison.

^s^FOCUS: family involvement, optimistic outlook, coping effectiveness, uncertainty reduction, and symptom management.

^t^iCBT: internet-delivered cognitive behavioral therapy.

^u^MR: Mission Reconnect.

^v^PREP: Prevention and Relationship Enhancement Program.

^w^OFPS: Online Family Problem-Solving Therapy.

^x^TBI: traumatic brain injury.

^y^WEQ: Website Evaluation Questionnaire.

^z^OSS: Online Satisfaction Survey.

^aa^SES: socioeconomic status.

^ab^CAPS: counselor-assisted problem-solving.

^ac^F-PST: family-problem-solving therapy.

^ad^TOPS: teen online problem-solving.

^ae^LI-IPV: low-intensity intimate partner violence.

^af^OR: odds ratio.

^ag^PACT: Parenting Acceptance and Commitment Therapy.

^ah^PERC: Prostate Cancer Education and Resources for Couples.

^ai^WiWi: Was It Worth It questionnaire.

### The Platforms

#### Overview

A total of 24 unique platforms were identified from the 85 studies. Table 2 shows the characteristics of the 24 platforms, including the intervention target; relationship targeted; duration of intervention participation; components designed for cocompletion, individual completion, and therapist engagement; any tailoring offered; and additional reported features.

Most interventions (14/24, 58%) were designed for cocompletion by couples, with some identified interventions for parent-child dyads (6/24, 25%), families (2/24, 8%), and caregiver–care recipient dyads (2/24, 8%). Given that it was expected that build characteristics might differ according to the population (eg, number of participating family members and their ages), platform results are grouped and reported by the relationship structure targeted by the platform (ie, couples, parent-child dyads, families, and caregiver–care recipient dyads).

Data from Table 2 are synthesized based on the features of the platforms and detail reported user engagement indicators. As platforms were included only in cases in which at least one study had demonstrated clinical efficacy of the intervention, mental health and relational outcomes are not reported in this table (and are, instead, indicated in Table 1).

**Table 2:** Characteristics of the platforms identified in the included studies, including name of the platform, relationship between the participants, platform purpose, duration of intervention participation, components that were completed in a self-paced manner, components completed together (cocompletion) or by individuals alone, practitioner engagement components, any tailoring provided, and additional key features.

Platform	Target relationship; intervention target; intervention duration	Self-paced components	Cocompletion versus individual completion	Practitioner engagement components	Tailored platform components and additional key features
4Cs:CRC^a^	Couples; patient–partner coping with cancer; 6 weeks	6 intervention sections including dyadic learning sessions, health information, cancer news, web-based counseling, sharing circle, and personal center	Content intended to be completed by couples together	Face-to-face or web-based synchronous counseling sessions delivered biweekly to revisit content and provide additional support (some study conditions)	Weekly reminders to complete web-based sessions
CA-CIFFTA^b^	Parent-child; treat behavioral problems and family conflict in young minority adolescents and their families; 12 weeks	4-6 computer-based modules; links to academic websites	Parents watched videos independently first, then rewatched with the adolescent; individual log-ins; role-appropriate videos	6-10 face-to-face sessions; fortnightly phone calls; asynchronous communication	Modular format for families to select content most relevant to the family’s clinical and cultural needs and preferences; custom links
Cool Kids Online	Parent-child; psychoeducation and CBT^c^-based anxiety management skills for children and their parents; 10 weeks	8 web-based lessons—first 6 released weekly and final 2 released biweekly	Web-based lessons completed together; parent trained as a “coach” for their child; additional web-based information provided to caregivers at the end of each lesson	Parents completed weekly phone calls with clinician—reinforce success, clarify questions, assist with barriers and skill implementation, reinforce practice, and normalize experience	Automated reminder emails—emails reinforced content, skill practice, and engagement
C-MBI^d^ for YBCSs^e^	Couples; relationship distress for couples where one member is a breast cancer survivor; 8 weeks	8 weekly, prerecorded videos delivered via the web; video links and reminders emailed to participants weekly	All videos watched together	Participants encouraged to email or call research staff regarding questions or content during participation	None
Couple HOPES^f^	Couples; relationship functioning when one partner has PTSD^g^; 8 weeks	7 web-based modules containing videos, exercises, and practice assignments completed sequentially	Videos and module exercises completed together; partners had separate, linked accounts where they independently completed assignments; assignment entries and scores could be seen by both partners	4 scheduled calls with a coach after modules 1, 3, 5, and 7 plus 1 additional call as needed; engagement and adherence facilitated through platform messaging; coaches’ role involved reviewing symptom change, reinforcing successes, enhancing motivation for engagement, and troubleshooting barriers	Automated feedback graph depicted reported symptom change over time; progress bar and module menu communicated and incentivized progress; web-based application and smartphone app
Couplelinks	Couples; relationship functioning after cancer diagnosis; 8 weeks	6 modules; each module begins with an informational component followed by instructions for interactive exercises; couples reflect after each module; additional articles and video resources available	Modules completed together	Asynchronous platform-based messaging; introductory telephone call and 2 brief “check-ins” to reinforce alliance and promote adherence	Additional noncompulsory content
eMB^h^	Couples; increase partner’s understanding of perinatal mood and anxiety disorders and therapeutic approaches to managing associated symptoms; 8 weeks	Recommended completion of 1 lesson per week in any order, with revisits as needed; psychoeducational modules containing YouTube videos, vignettes, interactive quizzes, homework, guided meditation, and downloadable resources	Participants could choose whether to complete separately or together	None	Could be completed in any order
Embers the Dragon	Parent-child; supporting emotional development and parental responses to child behavior; 8 weeks	Two 6-minute animated episodes and accompanying videos and activities	Parent and child watch the episodes and complete postvideo activities together; following the episodes, parents watch explanation videos	None	None
ePREP^i^	Couples; preventative intervention to enhance relationship satisfaction and mental health; 6 weeks	6 hours of web-based modules and approximately 1-2 hours of homework	Couples completed modules and homework together	Four 15-minute appointments with coach practicing skills; weekly reminder emails to complete content and links to resources	Computer based, could be completed from mobile or tablet
FOCUS^j^	Caregiver–care recipient (family); psychosocial health of patients with cancer and their family caregivers; 6 weeks	3 sessions delivered sequentially, with time to practice skills learned in between	Dyads completed the sessions together	Asynchronous “help” function that generated an email to the project director	Tailored, app-generated messages provided web links addressing the dyad’s specific concerns; offered a choice of tailored activities to complete between web sessions; tailoring based on baseline information provided
iCBT^k^	Families; family functioning when a child has an anxiety disorder diagnosis; 10 weeks	11 modules, including reading materials, film, animations, and illustrations	Parents worked on their modules first so that they could then work with the children; 7 modules aimed at parents only	Platform-based messages; tailored feedback after exercise completion; 3 telephone calls during treatment and additional ones as needed to clarify content, increase motivation, and solve problems	None
Military Family Foundations	Couples; military couples in the transition to parenthood; not specified	5 prenatal and 3 postnatal modules	Modules completed together	Email reminders sent to couples if they stopped engaging for >10 days	None
MindGuide Couples	Couples; preventative intervention centered on vulnerability to Korean middle adulthood depression, “Hwa-Byung,” and couple relationships; 5-7 weeks	4 modules over 16 sessions, maximum 60 minutes each; sessions included audio-recorded mindfulness activities, video lectures, practical tasks, and case-based scenarios	Modules 3 and 4 were joint sessions, including creating a shared vision; performed practical tasks together; modules 1 and 2 were completed individually	Coaching sessions after each module to promote participation via reflective dialogue and provide feedback on participants’ responses	None
MR^l^	Couples; relationship functioning when a member is a veteran with a history of deployment in a post-9/11 combat operation; 16 weeks	11 activities delivered via instructional videos, guided audio, and written manuals	Sessions on “Connecting with Partner” could be completed alone or together; the remaining sessions were completed independently	None	Accessible through website and mobile apps
Mother-daughter program	Parent-child; mother-daughter relationship quality and reduced risk of underage drinking; 10 weeks (4 weeks for the brief version)	9-14 modules; different adaptations were developed; animated characters portrayed the adolescent girl and her mother	Modules completed together; participants independently logged in to complete questions about content; participants could not advance until both mother and daughter had completed this	None	None
OFPS^m^ (including CAPS^n^ and TOPS^o^)	Families; family functioning when the child, adolescent, or teenager has a TBI^p^; 6 months	7-11 sessions; core sessions and additional supplementary sessions provided based on identified need; web-based content included problem-solving skills, video clips, exercises, and assignments	Website used by multiple family members together	Initial face-to-face session completed in the family’s home; telehealth session following web-based sessions to review exercises	Supplementary sessions provided based on personal need; family members selected their picture to indicate that they were present; when required, the platform would prompt particular family members to respond, and other times, the whole family was asked to respond together
OR^q^	Couples; relationship distress; 6 weeks (brief OR=2 weeks)	3 sections including video examples and psychoeducation	Content completed separately; couple completed guided conversation together at the end of each section	4 phone calls during the intervention; asynchronous chat feature	Tailored report on improvement provided; in some studies, automated tailored emails were provided
ParentSTRONG	Parent-child; adolescent boy domestic violence prevention intervention; 4 weeks	6 modules comprising 4-6 activities; parents and teenagers progress through alternate reality as avatars	After module 1, all modules are completed by the parent and child together; module 1 (introduction) completed by parents only	Staff could be contacted to troubleshoot technology	None
PACT^r^	Parent-child; emotional availability and parent and child adjustment when the child has cerebral palsy; 10 weeks (enforced break in the middle)	3 modules and a final review module after a short break	Some exercises were designed for individual completion	Fortnightly check-in (phone, SMS text message, or email) to monitor completion and check understanding of content	None
ParentWorks	Couples; father-inclusive parenting intervention; 4 weeks	5-8 modules	Participants accessed via a shared account; participants had the option to complete it independently	None	Feedback provided based on participant responses; formatted for mobile, laptop, and tablet viewing
PERC^s^	Couples; relationship distress following a prostate cancer diagnosis; 8 weeks	7 modules—5 core and 2 optional	Encouraged to view and complete everything together	None	Optional modules; users could select text- or audio-based slides depending on preference
Resilient Living	Caregiver–care recipient (family); building dyadic resilience skills for patients with stroke or brain tumor and their family caregivers; 8 weeks	4 web-based video modules and participant journal	Option to complete individually	Telehealth session before commencement of web-based modules	None
Web-based partnership support program	Couples; support intervention to prevent quality of life deterioration and reduce emotional distress in men undergoing fertility treatment; 2 weeks	30-minute self-paced content over 10 days	Watched information together; discussion between couples using the communication form; couples individually completed their communication form, which was subsequently used to guide their discussion	None	None
Web-based PREP^t^ program	Couples; couple relationship education for foster or adoptive parents; 1 week	4 chapters plus additional resources	Entire intervention completed together	None	None

^a^4Cs:CRC: Caring for Couples Coping With Colorectal Cancer.

^b^CA-CIFFTA: Computer-Assisted, Culturally Informed, and Flexible Family-Based Treatment for Adolescents.

^c^CBT: cognitive behavioral therapy.

^d^C-MBI: couple mindfulness-based intervention.

^e^YBCS: young breast cancer survivor.

^f^HOPES: Helping Overcome Posttraumatic Stress Disorder and Enhance Satisfaction.

^g^PTSD: posttraumatic stress disorder.

^h^eMB: mothers and babies online course.

^i^ePREP: computer-based Prevention and Relationship Enhancement Program.

^j^FOCUS: family involvement, optimistic outlook, coping effectiveness, uncertainty reduction, and symptom management.

^k^iCBT: internet-delivered cognitive behavioral therapy.

^l^MR: Mission Reconnect.

^m^OFPS: Online Family Problem-Solving Therapy.

^n^CAPS: counselor-assisted problem-solving.

^o^TOPS: teen online problem-solving.

^p^TBI: traumatic brain injury.

^q^OR: OurRelationship.

^r^PACT: Parenting Acceptance and Commitment Therapy.

^s^PERC: Prostate Cancer Education and Resources for Couples.

^t^PREP: Prevention and Relationship Enhancement Program.

#### Couples

##### Features of Platforms for Couples

###### Overview

Of the platforms requiring cocompletion, platforms designed for couples were the most common. A total of 58% (14/24) of the identified platforms were for couples. The intervention targets included relationship distress when a member has a cancer diagnosis (2/14, 14%); relationship functioning when a member has a cancer diagnosis (2/14, 14%), has posttraumatic stress disorder (1/14, 7%), or is a veteran (1/14, 7%); parenting-focused interventions, including a father-inclusive parenting intervention (1/14, 7%), education for foster and adoptive parents (1/14, 7%), and an intervention for military couples transitioning to parenthood (1/14, 7%); partnership support interventions for cases in which the male partner is undergoing treatment for infertility (1/14, 7%) or a member is pregnant (1/14, 7%); general relational distress (1/14, 7%); and preventative interventions to enhance relationship satisfaction and mental health (1/14, 7%) and reduce vulnerability to middle adulthood depression (1/14, 7%).

###### Structure and Duration of Engagement

Duration of participation varied from 1 to 16 weeks, with the most common duration being 8 weeks (5/14, 36%) followed by 6 weeks (4/14, 29%), including 1 intervention described as taking 5 to 7 weeks. The intended duration of 7% (1/14) of the interventions was not specified. One intervention offered a brief version that was completed by couples in 2 weeks as opposed to the 6-week full version. As per the inclusion criteria for this review, all interventions involved some web-based self-paced component completed on the platform. Most appeared to require at least weekly engagement, although it was not always specified or prescribed. One platform was designed such that participants could complete the intervention modules in any order but advised participants to access 1 module per week and complete all modules. For all the remaining interventions, it appeared that intervention content or modules were designed to be completed in a defined order and over a specified period.

###### Coparticipation and Contact With Practitioners

A total of 43% (6/14) of the interventions contained elements that were intended for individual completion (ranging from completing assessments to completion of entire sections of content), 50% (7/14) of the interventions required couples to cocomplete the whole intervention, and 7% (1/14) of the interventions gave participants the choice to complete some or all of the intervention together. In total, 57% (8/14) of the interventions included an element of practitioner engagement, including asynchronous platform-based messaging or scheduled synchronous counseling sessions.

###### Tailoring and Additional Features

Beyond personalization through contact with practitioners, 29% (4/14) of the platforms provided tailored content or options for personalization. A total of 14% (2/14) of the platforms provided supplementary content that could be accessed based on need, and 14% (2/14) of the platforms provided personalized feedback and reporting based on responses to questionnaires. In total, 29% (4/14) of the platforms specified that they were formatted for both web and mobile or tablet use, and 7% (1/14) of the platforms allowed participants to select either audio-enhanced or text-based presentation of content. Finally, 7% (1/14) of the platforms included an automated graph depicting reported symptom change over time and a progress bar to incentivize participation.

##### Reported User Engagement Indicators of Platforms for Couples

A total of 56% (48/85) of the studies examined the 14 couple-focused platforms. Of those 48 studies, 30 (62%) reported on user engagement indicators, including 23 (77%) studies that reported on satisfaction, feedback, usability, participant evaluation, feasibility, and acceptability and 18 (60%) that reported on completion rates and website use. The remaining 38% (18/48) of the studies did not report on any user engagement data or findings.

Measures used to collect participant satisfaction, feedback, usability, and evaluation varied. A total of 10% (5/48) of the studies administered the Client Evaluation of Services Questionnaire [126], and 15% (7/48) used the Client Satisfaction Questionnaire [127]. The remaining studies reported on satisfaction, feedback, and participant evaluation through nonvalidated measures. Satisfaction ratings were generally high across all studies.

The impact of video content on user engagement appeared mixed. Participants in 8% (4/48) of the studies provided feedback that the content and examples presented in the videos were helpful; however, in another 4% (2/48) of the studies, participants reported that the videos were unhelpful or that they negatively impacted engagement as they were not relatable, overly dramatized, or appeared outdated. In addition, participant qualitative feedback reported in another study suggested that outdated imagery and low-technology visualizations also negatively impacted engagement. Other factors that were reported to be important based on qualitative feedback included one study that reported on the structured nature of the intervention and reminder calls and another where participants reported that they were more likely to access audio-enhanced slides than text-based content. Feedback provided by participants in one study also noted that the flexibility of the web-based format facilitated engagement. However, in general, satisfaction, feedback, usability, and evaluation data were reported as average values on rating scales.

Reporting of completion rates and website use rates varied. They were reported as combinations of the following: the average number of participants who completed the entire intervention, the average number of modules or sessions completed by individuals or couples, the average time to completion, the number of discrete log-ins or page views, and the amount of time spent accessing the platform. Feasibility and acceptability data were reported similarly, with completion statistics often used as an indication of an intervention’s feasibility or acceptability. In addition, 5 studies reported on predictors of noncompletion, including 3 (60%) studies that reported higher levels of support from a practitioner as predictors of completion. The remaining 40% (2/5) of the studies reported on participant baseline characteristics as predictors of noncompletion.

Finally, 4% (2/48) of the studies on the same platform identified different couple “types” with regard to their enthusiasm and engagement (eg, “keen completers” or “stragglers”) and therapists’ role in engagement promotion. One study reported that higher levels of engagement (measured using participants’ correct responses to quiz questions) led to greater intervention effect on a number of clinical outcomes, and another found that those with the shortest time frame between commencement and completion (ie, completed the intervention faster) were more likely to be classified as “treatment responders” (identified by significant improvement on outcomes) at the postintervention assessment.

No studies of couple-based platforms identified build or design characteristics as moderators of intervention effect. No studies performed a formative evaluation of the platforms, and no studies reported design and build characteristics that enabled coparticipation beyond participant qualitative feedback.

#### Parent-Child Dyads

##### Features of Platforms for Parent-Child Dyads

###### Overview

Platforms designed for co-use by parent-child dyads were the second most common, accounting for 25% (6/24) of the platforms identified in this review. The intervention targets included behavioral problems and conflict in young minority adolescents and their families (1/6, 17%), emotional development and parental responses to child behavior (1/6, 17%), mother-daughter relationship quality and risk of underage drinking (1/6, 17%), adolescent male domestic violence prevention (1/6, 17%), emotional availability and parent-child adjustment when a child has cerebral palsy (1/6, 17%), and anxiety management skills and psychoeducation for parents and children (1/6, 17%). A total of 67% (4/6) of the platforms were developed for adolescents and a parent, 17% (1/6) were for young children aged 2 to 7 years and a parent, and 17% (1/6) were for children aged 7 to 12 years and their parents. In all cases, only 1 parent was asked to participate. In the following sections, we summarize the reported features of the platforms as detailed in the included studies.

###### Structure and Duration of Engagement

Duration of intervention use varied from 4 to 12 weeks, with the most common duration being 10 weeks (3/6, 50%). One intervention of a 10-week duration in total enforced an extended break in the middle of intervention engagement, and another offered a brief version of only 4 weeks (compared with the 10-week full version). Participation varied from once a fortnight to 2 web-based sessions or modules a week. A total of 83% (5/6) of the interventions appeared to involve completion in a structured manner following a predetermined order. One platform presented intervention content in a modular format that allowed participants to select the content that was relevant to their needs and cultural preferences in any order. However, the intended duration and number of modules accessed appeared to be prescribed.

###### Coparticipation and Contact With Practitioners

The amount and method of coparticipation varied greatly. A total of 33% (2/6) of the platforms required parents to watch the intervention content or preparatory materials before engaging with their adolescent child. In total, 33% (2/6) of the platforms required the parent to complete explanation videos or additional content following cocompletion with their young child. A total of 17% (1/6) of the platforms involved cocompletion of all intervention modules and independent completion of questions about content, with both the parent and adolescent required to complete these questions before the dyad could progress to the next module. Finally, studies on 17% (1/6) of the platforms reported that “some exercises” were designed for cocompletion but did not specify the extent of cocompletion.

A total of 67% (4/6) of the interventions included contact with a practitioner, whereas 33% (2/6) were entirely self-guided. One of those offering contact with a practitioner only offered this to parents and not the participating child. A total of 33% (2/6) of the interventions included scheduled sessions with a practitioner to discuss content, with 17% (1/6) also supporting asynchronous communication with a practitioner via the platform. Finally, in 17% (1/6) of the interventions, participants could contact practitioners via the platform for technical troubleshooting as required.

###### Tailoring and Additional Features

In total, 17% (1/6) of the platforms allowed participants to select content based on their clinical and cultural needs. Content was selected from a list of available modules, although the process through which the dyads selected this content was not described. This same platform offered dyads links to external sources of information based on their responses to questionnaires.

##### Reported User Engagement Indicators of Platforms for Parent-Child Dyads

A total of 14% (12/85) of the studies evaluated 6 different interventions designed for use by parent-child dyads. Of those 12 studies, 5 (42%) reported on user engagement indicators, including completion or fidelity (4/5, 80%) and satisfaction or acceptability (3/5, 60%). The remaining 58% (7/12) of the studies reported on mental health or relational outcomes and did not report on user engagement indicators.

The 33% (4/12) of the studies reporting on completion or fidelity documented the number of participants who completed the entire intervention as prescribed. One study also reported on the average time it took participants to complete the intervention, and another reported on the number of dyads who accessed all sessions and received calls from a practitioner. No studies reported on participants’ interaction with the platform or any predictors of noncompletion.

All studies reporting on satisfaction and acceptability did so using nonvalidated measures. Mean satisfaction ratings were high. One study asked participants to indicate how easily they found time to complete the activities together, with a mean rating of 3.04/5 (SD 0.37) for daughters and a mean rating of 3.24/5 (SD 0.33) for mothers. In no study did the satisfaction and acceptability data distinguish between platform and intervention satisfaction.

No studies on parent-child interventions identified platform build or design characteristics as moderators of intervention effect. No studies performed formative evaluations of the platforms, and no studies reported on design and build characteristics that enabled coparticipation.

#### Families

##### Features of Platforms for Families

###### Overview

Among the 24 platforms, 2 (8%) designed for cocompletion by families were identified. The intervention targets included family functioning when a child has an anxiety diagnosis (1/2, 50%) and family functioning when a child, adolescent, or teenager has a traumatic brain injury (1/2, 50%). Both platforms were intended for use by a child, adolescent, or teenager with a presenting clinical concern and any family members, including parents and siblings. Though siblings and other family members were invited to participate, the studies detailed outcomes and engagement for a single parent and child only.

###### Structure and Duration of Engagement

Intervention participation on one platform extended for 10 weeks over 11 web-based chapters, and the other delivered 7 to 11 sessions over 6 months. Both were designed for sequential completion of module content.

###### Coparticipation and Contact With Practitioners

One platform asked family members to complete the entire intervention together. The other asked parents to complete sections themselves before working with their children on a small number of modules intended for cocompletion. Both included scheduled telehealth sessions with a practitioner during intervention participation. In addition, one platform also included a platform-based message system for contacting practitioners asynchronously. In this same platform, practitioners also provided reports to participants following exercise completion.

###### Tailoring and Additional Features

One platform provided no tailoring beyond engagement with and feedback provided by practitioners. The other platform included supplementary sessions that could be completed by families should they wish to. In addition, this platform supported cocompletion by asking family members to select their picture when they were present. The platform would then either prompt individual family members to respond or ask all family members to respond together.

##### Reported User Engagement Indicators of Platforms for Families

A total of 27% (23/85) of the studies examined the 2 family-based interventions. Of these 23 studies, 16 (70%) reported user engagement indicators including satisfaction and ease of use (n=9, 56%); completion rates, compliance, adherence, and website use (n=13, 81%); and feasibility (n=2, 12%). The remaining 30% (7/23) of the studies did not report on satisfaction, completion, or feasibility data or findings.

Of the 9 studies reporting satisfaction and ease of use, 6 (67%) used an adaptation of the Website Evaluation Questionnaire [128] to measure participant satisfaction with the intervention. The remaining 33% (3/9) of the studies administered nonvalidated measures developed for the studies. Satisfaction ratings were high across all studies. In 33% (2/6) of the studies in which the Website Evaluation Questionnaire was administered, participants were asked to rate the website’s ease of use, generally reporting that the website was “moderately easy” to “easy” to use. Participants in one study reported a preference for meeting in person. Other than this, satisfaction ratings either were relevant to content or did not distinguish between platform and intervention satisfaction.

Completion rates, compliance, adherence, and website use were all reported as combinations of the following: the number of participants who completed the entire intervention, the average number of modules completed, time spent on the platform, and the number of families who completed supplemental sessions. Feasibility was reported similarly, with one study also reporting that families were able to complete all sessions without practitioner assistance. In addition, one study reported on number of sessions completed as a predictor of symptom change (with inconsistent effect), and another reported on participant characteristics at baseline as predictors of completion.

A total of 13% (3/23) of the studies also measured participants’ technology use and comfort with technology before the commencement of the intervention and examined this as a predictor of intervention effect. Results were inconsistent. In addition, one study identified whether participants’ preference for treatment modality before the intervention, that is, face-to-face, web self-paced, or therapist-guided modality, impacted treatment outcomes. It was found that adolescent treatment preference was significantly related to attrition, but there were no other links with treatment effect or satisfaction.

No studies on family-based platforms identified build or design characteristics as moderators of the intervention effect. No studies performed a formative evaluation of the platforms, and aside from one study describing how participants identified that they were present, no studies reported design and build characteristics that enabled coparticipation.

#### Caregiver–Care Recipient (Family) Dyads

##### Features of Platforms for Caregiver–Care Recipient Dyads

Among the 24 platforms, 2 (8%) were identified for family caregiver–care recipient dyads. The targets of the interventions on the platforms included dyadic resilience for patients with stroke or brain tumor and their family caregivers (1/2, 50%) and the psychosocial health of patients with cancer and their family caregivers (1/2, 50%).

###### Structure and Duration of Engagement

One of the 2 platforms involved intervention participation over 6 weeks, with 3 sessions delivered sequentially. The other platform contained 4 web-based modules and a participant journal completed over 8 weeks. While not explicitly reported, it appeared that this platform also required sequential completion of intervention content.

###### Coparticipation and Contact With Practitioners

Both platforms were designed to be completed by members of the dyad together; however, one had the option of completing the entire intervention independently if desired. One platform contained an asynchronous help function that generated an email to the project director. The other included a telehealth session before commencement of the web-based component.

###### Tailoring and Additional Features

One platform contained several tailored elements, whereas the other did not offer any personalization. Tailoring included platform-generated messages that provided web links addressing the dyad’s concerns and supplementary activities offered between web sessions. Both were generated from self-reported baseline information.

##### Reported User Engagement Indicators of Platforms for Caregiver–Care Recipient Dyads

The 2 interventions were each evaluated in 1% (1/85) of the studies. Both studies reported on satisfaction, and one reported on feasibility, with both reporting high satisfaction ratings. One study reported that there were no adverse effects of participants completing the intervention on the web-based platform together, and the other identified the length of the modules and the ability to complete the intervention in the users’ own time as facilitators to use. In one study, participants noted that finding time to complete the intervention as a dyad was sometimes challenging. Where feasibility was reported, the study found lower enrollment rates than those for previous in-person randomized controlled trials but higher retention rates.

Neither study identified build or design characteristics as moderators of intervention effect. No study performed a formative evaluation of the platforms, and neither reported on design nor build characteristics that enabled coparticipation.

## Discussion

### Principal Findings

This review details build, design, and user engagement characteristics of platforms that enable cocompletion of clinical interventions by related people. To distinguish effective platform contributors to engagement from elements pertaining to intervention content, we selected only those platforms housing interventions of established clinical efficacy (ie, previously reported significant improvement of at least one mental health or relational outcome). Some common design features were identified; however, in contrast to expected findings, specific design characteristics enabling cocompletion were rarely reported, and evidence for engaging families was underexplored.

### Common Platform Features

This review identified platform design features that were common across the included studies. Regardless of the relationship targeted, most platforms delivered a structured intervention that required engagement over a prescribed duration with content completed sequentially. A total of 8% (2/24) of the platforms allowed participants to access content in a nonsequential manner, and a handful (4/24, 17%) offered supplementary content based on identified need. Retention rates remain low for DMHIs [29], and there are further complexities when family members participate together [50]. As such, consideration might be given to ways in which families’ time on the platform can be optimized.

Single Session Thinking is one process through which therapists treat each encounter as if it were the sole session, encouraging the participants to make the most of the time [129]. Adaptation to web delivery of family therapy sessions already holds promise [130], and digital single-session interventions have been trialed in college student settings with positive preliminary findings [131-133]. Therefore, there is emerging evidence suggesting that Single Session Thinking principles could be readily applied to DMHIs, mimicking single, stand-alone sessions that address the family’s present needs as they identify them. Check-in prompts and invitations to return as needed could be automated from the platform to encourage return visits as required or desired by the family. A platform designed to deliver content in this way would likely reduce the burden on families and provide greater flexibility in how they access content.

Minimal tailoring was offered in 29% (7/24) of the DMHIs identified in this review, providing more or less the same intervention to all participants. A total of 67% (16/24) of the interventions included interaction (either synchronous or asynchronous) with a practitioner. Evidence for personalized mental health care is growing rapidly, acknowledging the complexity and diversity of individuals and families [134,135]. Higher levels of engagement are reported for guided interventions (ie, those where participants have some contact with a practitioner) than for self-guided interventions; however, incorporating human contact can be costly and can limit the flexibility and accessibility associated with DMHIs [136]. Research suggests that, compared with targeted or generic feedback, personalization can be used to improve engagement and subsidize personal contact and contributes to positive attitudes toward a DMHI [134,135]. Beyond this, several studies included in this review (9/85, 11%) identified baseline characteristics that moderated participants’ responses to the intervention. These included characteristics such as age, relationship status, and previous comfort with technology. Understanding how baseline measures might impact participants’ ability or desire to engage with platforms and providing options for personalization accordingly would likely result in greater engagement. A family-based platform might include tailored design options such as color and font choices, preferences for video- or text-based content, and preferences for receipt of prompts and reminders. In addition, if children are present, families could have the option to access content that has been adapted for younger readers. In a world where artificial intelligence is supporting personalization across the internet, it would be remiss not to consider personalization in family- and relational-based DMHIs.

### Platform Features for Enabling Cocompletion

By their nature, computers and mobile devices are designed for use by individuals. Given obvious complexities involved in having multiple people participate in a web-based intervention together, it was expected that platforms designed for such use may contain features for enabling cocompletion across the life span. It was also expected that the way in which participants engage may differ from that in platforms designed for individual use. This could include considerations about privacy of individual participants’ data, methods for encouraging participants to work together, and design choices to allow all participants to contribute to activities. One platform requested participants to select their image when they were in attendance, and this was then used to prompt individuals to respond and participate in activities. Other than this, no study identified platform characteristics that were included to specifically enable cocompletion. In general, studies detailed participants’ engagement with the intervention but not with the platform. Reporting on platform engagement might include details on how participants navigated the interface, how they identified and accessed content, or the modes through which content was delivered. On the other hand, intervention reporting was found to delve into factors such as attrition rate and measurement completion. Crucially, it is important to distinguish between intervention trial attrition (ie, dropout or loss to follow-up) and platform disengagement (ie, nonuse attrition), as recommended in a previous review [137]. These 2 forms of attrition are influenced by distinct factors [138,139], and failure to differentiate between them could potentially lead to misinterpretation of platform engagement dynamics.

It was also expected that studies would provide insights into the build and design considerations related to individual user privacy and safety within a shared web-based space. This encompasses considerations such as determining when an individual’s information can or should be shared with other members of the family and effectively identifying and responding to safety risks. From our perspective, these design aspects are essential considerations when developing a family-based DMHI. However, none of the studies identified in this review reported or discussed how they tackled or addressed these privacy and safety considerations. To further ensure the adequate addressing of not only these concerns and anticipate other potential considerations, rigorous co-design processes are essential. This co-design strategy would significantly contribute to the refinement of family-based DMHIs, ensuring that they meet the nuanced needs of users.

### Engagement With Practitioners

The varied nature of engagement with guided tools (ie, involving interaction with practitioners, structured sessions, and feedback loops) stands in stark contrast to the self-guided use and consistent participation characterizing engagement with tools lacking contact with practitioners. Recognizing challenges intrinsic to self-guided tools, such as user motivation and adherence, becomes paramount, particularly given that the absence of practitioner involvement is likely to make the sustainability of user interest more demanding. The role of technology in promoting engagement with practitioners is multifaceted, encompassing communication facilitation through asynchronous methods and data-driven insights that enhance personalized interactions. Moreover, exploring hybrid models and incorporating periodic check-ins or teletherapy sessions within self-guided platforms presents a promising balance between autonomy and professional support.

Addressing challenges in technology engagement involves prioritizing user-centered design; integrating behavioral science principles; and leveraging feedback mechanisms, either automated or through clinician input, to ensure continuous support and guidance. Looking forward, suggested avenues for future research are many, including the long-term effectiveness of guided and self-guided tools, understanding the impact of different engagement strategies, and developing sophisticated technology-assisted therapeutic approaches.

### Evidence for Enabling Cocompletion

We faced constraints in reporting evidence on platform features that engaged and enabled cocompletion by families because no study conducted a direct evaluation of the platform design. This limitation hindered our ability to provide comprehensive insights into the effectiveness of features promoting cocompletion among participating family members. In addition, while several studies (13/85, 15%) evaluated practitioner support, family member coparticipation, population characteristics, and baseline scores on mental health or relational measures as moderators of intervention outcomes, no study evaluated design features as potential moderators of intervention outcomes.

Of the 85 included studies, 66 (78%) reported on user engagement indicators. Of those, most (48/66, 73%) used custom, nonvalidated measures, and the remaining studies used validated measures that were intervention specific and gave no information about platform engagement. Given this measurement heterogeneity, little is possible by way of cross-study comparison. In addition, without evaluation of platform design strategies, no conclusions can be drawn about enabling or disabling features. The capacity for real-world translation and understanding of how to overcome known barriers is constrained.

### A Need for Cohesive Platform Evaluation and Reporting

Platform user experience design, including ease of use, navigation, screen layout, readability, gamification, feedback, and attractiveness, plays a large role in a participant’s perception of and engagement with a website and, ultimately, a site’s usability [29,140,141]. In addition, individual participant characteristics such as age, literacy level, level of disability, and mental health conditions may impact their engagement with and ability to use a platform as designed. When a family presents on a web-based platform, more than one person’s needs must be catered to.

There is a lack of consensus and shared understanding of how to usefully conceptualize and measure engagement with and accessibility of digital mental health platforms [35,36]. This variability is not unique to the context of family-based mental health platforms, with reviews of engagement in digital mental health reporting similar heterogeneity [35,134,142]. Studies tend to report on measures such as completion or attrition rates, usability, user satisfaction, acceptability, and feasibility as indicators of how well the application engaged users. Often, these data are self-reported. Given the high attrition rates for self-guided platforms [102] and additional complexities involved in requiring family members to cocomplete activities [50], understanding platform characteristics that enable co-use and promote engagement is vital to informing the future development of such platforms. There is limited direct evidence to support practitioners, developers, and designers in understanding why engagement levels remain low, and there remains a limited understanding of how to design a DMHI to optimize engagement for families.

Assessment of user engagement indicators such as completion data alone is likely insufficient to measure how well a platform engaged its users. For example, reporting on duration of participation and sessions completed neglects factors impacting how families navigate the website, such as interface design and organization, and user characteristics. Analysis of platform use patterns and baseline characteristics in addition to these completion statistics would provide greater insights into how families engage with a platform. Formative as opposed to summative evaluations of usability are conducted to inform the redesign and improvement of a web interface. Formative evaluations consider multiple factors and involve building a deep understanding of user perceptions and use patterns of platforms. In addition to self-reported measures and completion rates, formative evaluations often also consider website analytics such as bounce rate, pages per session, top exit pages, and the pathways that users take to get to pages where they ultimately spend most of their time. It is a recommendation of this review that formative evaluations of web-based mental health platforms become common practice for DMHIs, particularly for novel and complex applications such as family-based platforms.

Finally, a systematic review of evaluations of usability of mobile mental health technologies [143] recommended closer collaboration between health care and computer science experts when evaluating DMHIs, suggesting that this would increase the quality of interpretation of the evaluation. A summary of learnings from the *ParentWorks* trial identified an expected benefit of having involved a web agency during the early stages of content translation to optimize user experience [144]. An interdisciplinary approach might enhance knowledge sharing, too, through detailed reporting of DMHI design decisions and their interactions with platform elements and clinical outcomes.

Clearly, there remains a need for coherent reporting and evaluation practices in the field to inform guidelines and policy on effective strategies for engaging families on the web in mental health–related interventions. Until rigorous co-design with families and an interdisciplinary approach between content experts and user experience designers is taken to formative evaluations, the growth and expansion of efficacious mental health platforms for family use will lag.

### Study Strengths, Limitations, and Future Research Directions

This study represents the first of its kind. Using a replicable search strategy over 4 periods, we synthesized in this study the state of the published evidence regarding platform design and build characteristics enabling successful engagement of related parties with digitally delivered mental health interventions. Given that gray literature was not searched for this review, it is possible that emerging evidence for new multiuser digital platforms was missed. Our findings are limited by the technical reporting of the studies. Principally, many studies did not provide details about their platform build or the way in which participants engaged with the platform, including whether coparticipation was expected. Where this information was not provided, the study authors were contacted, and websites were searched to retrieve the relevant information. It is likely that examination of some relevant functionality was precluded when this information was not provided or was insufficient.

Many studies (24/85, 28%) explicitly excluded participants when those other than the identified person had a mental illness. Whether through caregiving burden, stigma, or familial shared conditions, it is rare for a family presenting for therapy to have only 1 member experiencing mental health stress or significant challenges [145,146]. Given the potential of these platforms to aid family therapy, further research with families in which multiple members experience stress or mental health challenges is needed. Until then, it is difficult to generalize the evidence reported in this review to the real-world experience of families who may present for family therapy.

In addition, diversity in populations was limited, with most studies including White, heterosexual, and middle-class participants. There was a lack of evidence from low- and middle-income countries (LMICs), with all studies conducted in more high-income countries. The technological experiences and needs of families in LMICs will likely vary significantly from those in more high-income countries given, among other factors, the varying degree of ease of access to technology. Digital interventions have the potential to expand reach and access to services; however, until participants from LMICs are included in studies of digital platforms for families, findings cannot be generalized to these populations and ultimate reach will be limited.

As this is a new and novel field, language and terminology are still being defined, and means of measuring and defining engagement and feasibility are not well established [29]. Of the included studies, 52% (44/85) were published in the last 5 years, reflecting rapid developments in technology and associated applications.

### Conclusions

While there is emerging evidence suggesting that DMHIs are clinically effective, there remains a large evidence gap in the literature on the extent to which platform-specific design and build elements may also contribute to timely access, user experience, safe cocompletion by family members, and clinical outcomes. In the service of improved mental and relational health outcomes, our findings point to a significant opportunity for meaningful cross-disciplinary research, development, and evaluation of family-based mental health platforms. Findings from the next era of research will be central to enabling policy and practice advancements in equitable access to effective mental health care support for families.

## Appendix

Multimedia Appendix 1 Excluded studies and platforms.

Multimedia Appendix 2 Search strategies.

## Abbreviations

DMHI: digital mental health intervention
LMICs: low- and middle-income countries
PRISMA: Preferred Reporting Items for Systematic Reviews and Meta-Analyses
PRISMA-ScR: Preferred Reporting Items for Systematic Reviews and Meta-Analyses extension for Scoping Reviews

## Data Availability

All data generated or analyzed during this study are included in this published article (and its supplementary information files).
