# Supplementary material for: Design of Digital Mental Health Platforms for Family Member Cocompletion: Scoping Review
Source: J Med Internet Res. 2024 Jul 3;26:e49431. doi: 10.2196/49431 (PMC11255536; doi:10.2196/49431)
Supplement: Multimedia Appendix 1 [file jmir_v26i1e49431_app1.docx]

# Appendix 1 – Excluded Studies and Platforms

Table 4 Studies not reporting significance in clinical outcomes

| **Platform/Study/Country** | **Study Characteristics** | | **Measures/Outcomes** | | |
| --- | --- | --- | --- | --- | --- |
|  | **Design; comparator** | **Population; sample; attrition** | **Measurement constructs/outcomes**  **Relational; individual** | **User engagement indicators** | **Reported findings** |
| e-MTSD  [148]  USA | Pilot feasibility trial; text reminders (TR)/ action planning (AP)/ text reminders + action planning (TRAP)/ low adherence support (LA) | Mother-adolescent dyads where the mother has experience intimate partner violence; 101 dyads; 17% | Self-reported program impact post-intervention | 1. Acceptability  2. Uptake, use, duration of access  3. Se | 1. 90% agreed with indicators of program acceptability; fewer adolescents than mothers endorsed “this module kept my attention”; respondents in TR and TRAP conditions indicated text messages were helpful reminders and 92% indicated it was an appropriate number of messages; 76% of AP and TRAP participants completed action plan, 58% reporting it was very helpful.  2. 1 dyad did not access program; 5% did not complete any module; 75% completed all modules; completion rates were highest for TRAP condition (84%), followed by LA (73%), AP (72%) and TR (68%); Med time to completion 146 mins; |
| FAMOCA  [149]  Switzerland | RCT; treatment as usual plus resources | Parent diagnosed with cancer for the first time, their partner and child(ren) between 3-18 years ; 63 participants from 22 families (34 parents, 29 children); 46% at post-treatment and 59% at follow-up | Family functioning; Children’s quality of life; Children’s behavioural-emotional adjustment | 1. Use of the website  2. Evaluation | 1. Participants spent average of 32 minutes on the website  2. Families appreciated variety of information provided and freedom to work independently; children and adolescents liked the active parts of the program; most families reported there was too much text to read |
| Spelfri.se  [150]  Sweden | RCT; treatment as usual | Individuals who met the criteria for problem gambling and a concerned significant other; 18 dyads; Intervention 35%; control 37.5% | Pathological gambling; Net losses on gambling; Depression; Anxiety; Alcohol consumption | 1. Experience of treatment | 1. Intervention gamblers rated satisfaction 4.8/5 on average; intervention CSOs rated satisfaction 4.8/5 on average; all intervention participants would recommend the program |

^a^ indicates intervention significantly changed (single-arm) or superior to comparator in hypothesized direction at post-intervention; RCT = randomised controlled trial; IRC = internet resource comparison; QoL = quality of life; M = mean/average; SD = standard deviation

Table 5 Platforms excluded based on lack of clinical efficacy

| **Platform** | **Target relationship; intervention target; intervention duration** | **Online self-paced component(s)** | **Co-completion vs individual completion** | **Practitioner engagement component(s)** | **Tailored platform component(s); additional key features** |
| --- | --- | --- | --- | --- | --- |
| **e-MTSD** (moms for Teens and Safe Dates) | Parent-child; Increase mother-adolescent positive communication about healthy and unhealthy relationships and reduce the adolescent’s risk of experiencing dating violence; 6-weeks | 6 online modules completed sequentially including information and interactive activities | 5 program modules completed together; Mothers independently complete prep module. One study condition had mothers create an action plan, outlining their plan for completing and overcoming engagement barriers. | None | Some study conditions received tailored message at fixed intervals (based on whether they were on track); Reminders texts; tech support provided via text |
| **FAMOCA** (Family Online Counselling for Families with Parental Cancer) | Families; Improving child and parental adjustment and family functioning where a parents has cancer; 16-weeks | 4 online modules | Most modules were designed for parents to work with their younger children (3-11); Age-specific modules for children and adolescents; adolescents generally completed independently; some elements were designed for young children to complete independently | Monthly therapist contact to provide feedback, answer questions and unlock content | Presentation varied for age range, e.g. young children listened to stories while older children watched film clips |
| **Spelfri.se** | Couples; To inform and support gambling addicts and their relatives; 12-weeks | 10 modules | Several exercises in each module were designed for co-completion | Scheduled telephone and email support from therapist | None |
