# Supplementary material for: Design of Digital Mental Health Platforms for Family Member Cocompletion: Scoping Review
Source: J Med Internet Res. 2024 Jul 3;26:e49431. doi: 10.2196/49431 (PMC11255536; doi:10.2196/49431)
Supplement: Multimedia Appendix 2 [file jmir_v26i1e49431_app2.docx]

# Appendix 2 – Search Strategies

*Medline (OVID)*

| **Concept (combined with AND)** | **Terms (combined with OR)** |
| --- | --- |
| Online | (computer based or web based or digital or computer assisted or computeri*ed or internet* or online or "on line" or technolog*) adj4 (program* or intervention* or therap* or treatment* or psychoeducat* or educat* or assessment*) |
| Mental/relational health | ((mental or relation*) adj4 (health or function* or satisfaction or distress)) or psychiatr* or psycholog* or "family relation*" |
| Population | parent* or famil* or couple* or dyad* or kin* or sibling* |
| Limits | English only  2002 – current |

*Embase (OVID)*

| **Concept (combined with AND)** | **Terms (combined with OR)** |
| --- | --- |
| Online | (computer based or web based or digital or computer assisted or computeri*ed or internet* or online or "on line" or technolog*) adj4 (program* or intervention* or therap* or treatment* or psychoeducat* or educat* or assessment*) |
| Mental/relational health | ((mental or relation*) adj4 (health or function* or satisfaction or distress)) or psychiatr* or psycholog* or "family relation*" |
| Population | parent* or famil* or couple* or dyad* or kin* or sibling* |
| Limits | English only  2002 – current |

*PsycINFO (OVID)*

| **Concept (combined with AND)** | **Terms (combined with OR)** |
| --- | --- |
| Online | (computer based or web based or digital or computer assisted or computeri*ed or internet* or online or "on line" or technolog*) adj4 (program* or intervention* or therap* or treatment* or psychoeducat* or educat* or assessment*) |
| Mental/relational health | ((mental or relation*) adj4 (health or function* or satisfaction or distress)) or psychiatr* or psycholog* or "family relation*" |
| Population | parent* or famil* or couple* or dyad* or kin* or sibling* |
| Limits | English only  2002 – current |

*Web of Science*

((TS=(("computer based" or "web based" or digital or "computer assisted" or computeri?ed or internet* or online or "on line" or technolog*) NEAR/4 (program* or intervention* or therap* or treatment* or psychoeducat* or educat* or assessment*))) AND TS=(((mental or relation*) NEAR/4 (health or function* or satisfaction or distress)) or psychiatr* or psycholog* or family relation*)) AND TS=(parent* or famil* or couple* or dyad* or kin* or sibling*)

English only

2002 – current

*CINAHL (Ebscohost)*

| **Concept (combined with AND)** | **Terms (combined with OR)** |
| --- | --- |
| Online | (computer based or web based or digital or computer assisted or computeri*ed or internet* or online or "on line" or technolog*) N4 (program* or intervention* or therap* or treatment* or psychoeducat* or educat* or assessment*) |
| Mental/relational health | ((mental or relation*) N4 (health or function* or satisfaction or distress)) or psychiatr* or psycholog* or "family relation*" |
| Population | parent* or famil* or couple* or dyad* or kin* or sibling* |
| Limits | English only  2002 – current |
